# Cyanobacterial Community Structure and Isolates From Representative Hot Springs of Yunnan Province, China Using an Integrative Approach

**DOI:** 10.3389/fmicb.2022.872598

**Published:** 2022-04-25

**Authors:** Nitin Keshari, Yang Zhao, Sudipta Kumar Das, Tao Zhu, Xuefeng Lu

**Affiliations:** ^1^CAS Key Laboratory of Biofuels, Shandong Provincial Key Laboratory of Synthetic Biology, Qingdao Institute of Bioenergy and Bioprocess Technology, Chinese Academy of Sciences, Qingdao, China; ^2^Shandong Energy Institute, Qingdao, China; ^3^Qingdao New Energy Shandong Laboratory, Qingdao, China; ^4^Centre of Excellence in Integrated Omics and Computational Biology, Utkal University, Bhubaneswar, India; ^5^College of Life Science, University of Chinese Academy of Sciences, Beijing, China; ^6^Laboratory for Marine Biology and Biotechnology, Qingdao National Laboratory for Marine Science and Technology, Qingdao, China

**Keywords:** hot springs, cyanobacteria, 16S rRNA amplicon sequencing, phenotype, phylogenetics

## Abstract

Cyanobacteria from the representative hot springs of Yunnan Province, China are explored for their diversity and community composition following an integrative approach of cultivation-independent and -dependent studies and further isolation of potential taxa for future biotechnological perspective. 16S rRNA amplicon sequencing of microbial mats in these hot springs with temperature ranging from 38 to 90°C revealed Cyanobacteria and Proteobacteria constituting a bounteous portion of the bacterial community. The combined approach of 16S rRNA amplicon sequencing and phenotypic analysis revealed the diversity of cyanobacteria (a total of 45 genera). Out of these, a total of 19 cyanobacterial taxa belonging to 6 genera and 10 species were isolated as individuals with the possibility of biotechnological utilization. These isolates were subjected to a thorough morphological study and molecular characterization using 16S rRNA gene sequencing for identification and understanding their phylogeny. The identity and phenotypic and genotypic characteristics of 7 cyanobacterial isolates are not identical to any known cyanobacterial species, generating scope for future taxonomic novelties. Preliminary experiments based on high-temperature (50°C) cultivation showed that most of the isolates were thermotolerant and suggested for their high biotechnological usage potential.

## Introduction

Hot springs or thermal springs are natural geological phenomena related to the expulsion of hot water (at or above 36.7°C) from the earth (Pentecost et al., 2003). These are stabilized ecosystems with highly constant abiotic components like temperature, pH, and ionic composition, which also inhabit consistent biotic resources. This consortium of microorganisms not only is adaptive to thermal extremity but also persisted through a long period of time, which can provide an insight into microbial evolution (McGregor and Rasmussen, 2008). The micro-vegetation in the thermal springs is dominated by cyanobacteria when the pH value is beyond 6 and temperature is below 74°C (Brock, 1967). There were several documentations of cyanobacteria that can survive at higher temperatures (Castenholz, 1969; Singh et al., 2018). Hot springs harbor potential thermotolerant strains for biotechnological applications. Mat-forming thermal cyanobacteria have the ability to produce a wide range of secondary metabolites with bioactive properties (Brito et al., 2015; Derikvand et al., 2017), which could be the rationality behind the isolation, and to enrich the culture collection of thermophilic cyanobacteria.

Hot springs of Yunnan Province are located in the Indo-Burma range, which is well known for widespread geothermal activity as a result of the complex geological history of the region (Hedlund et al., 2012; Hou et al., 2013). Extensive volcanism resulted in geothermal areas throughout Tengchong town, leading to many active hot springs (Du et al., 2005). Hot springs of Tengchong can be considered as biodiversity hotspots and compared with microbial diversity inhabiting other geothermal areas around the world, such as Yellowstone National Park, United States (Fournier, 1989), Japan (Yoshida, 1991), and Kamchatka, Russia (Kyle et al., 2007). Both cultivation-dependent and -independent examinations as well as biotechnological potential of thermophilic microbes (bacteria and archaea) from the hot springs of Yunnan Province were studied (Hedlund et al., 2012). However, a comprehensive census of the cyanobacterial communities, especially through next-generation sequencing (NGS) of hyper-variable regions of the 16S rRNA gene and isolation of potential strains for biotechnological applications, is still lacking.

Cyanobacteria were traditionally divided into five subsections based on their morphological complexity. Cyanobacteria of subsections I and II are unicellular while those of subsections III–V form various filamentous morphologies. Cells of subsection I divide by binary fission, whereas those of subsection II can also reproduce through multiple fissions in three planes to generate baeocytes (small, easily dispersed cells). Strains in subsection III have only vegetative cells, but in subsections IV and V, vegetative cells can differentiate into heterocysts (specialized nitrogen-fixing cells) or akinetes (resting cells that survive environmental stresses). In addition, strains in subsection V are further distinguished by branching patterns of their filaments (Tomitani et al., 2006; Shih et al., 2013).

To enrich the current state of the knowledge, the cyanobacterial diversity in the biofilms of different hot springs of Yunnan Province was explored for the first time. The objective of the study addresses four main questions: (1) What is the cyanobacterial composition in these thermal microbial mats? (2) How is the morphological diversity related to the meta-genomic diversity? (3) Could combined phenotypic and meta-genomic approach help for better understanding of cyanobacterial diversity? (4) Could the hot spring cyanobacterial isolates truly be able to endure heat stress under laboratory conditions? We have conducted both cultivation-independent (meta-genomic and traditional microscopic study together) and -dependent studies that offer a comprehensive assessment of microbial composition and isolation of novel cyanobacteria to explore their properties, respectively. Thus, the findings will not only expand our current knowledge of thermal microbiome in China but also provide clues and cyanobacterial strain resources for their biotechnological prospective.

## Materials and Methods

### Study Sites and Sampling

**FIGURE 1:**
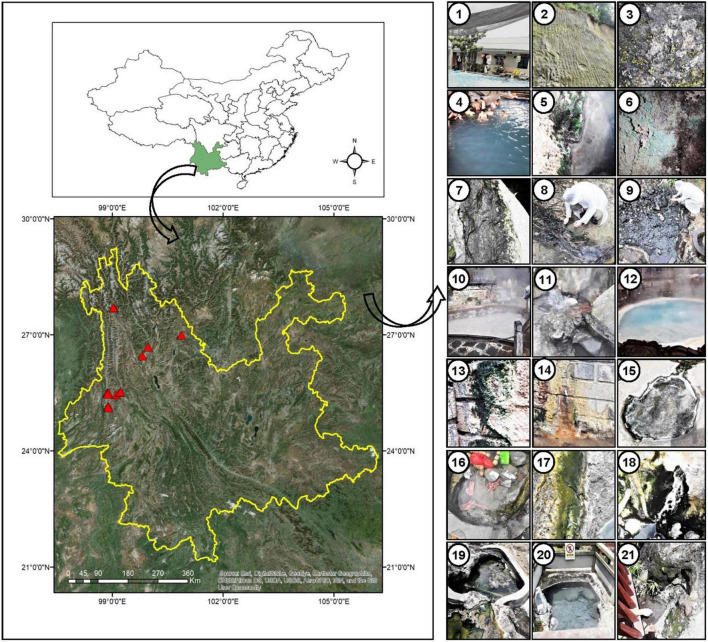
Location of sampling sites (marked with red triangles) in Yunnan Province, China. Geographic coordinates were determined using a Garmin Etrex GPS. Digital Elevation Model (DEM) data information was downloaded from Resource and Environment data cloud Platform of China (http://www.resdc.cn/). Pictures in the right panel indicate the surrounding sampling environment of the cyanobacterial biofilms in different hot springs, 1: Dongshan-001, 2–3: Huangpo-002 (soil), 4–7: Huangpo-003 (4 samples), 8: Rehai (Hamazui)-004, 9: Rehai (Hamazui)-005, 10: Rehai (Yanjingquan)-006, 11: Rehai (Zhenzhuquan)-007, 12: Rehai (Dagunguo)-008. 13–14: Rehai (Dagunguo)-009, 15: Banglazhang-011, 16–17: Banglazhang-012, 18: Banglazhang-013, 19: Banglazhang-014, 20: Banglazhang-015, 21: Banglazhang-016.

The sampling sites are under the Indo-Burma biodiversity hotspot, located near the China-Myanmar border (Figure 1) in Tengchong town (Dongshan, Huangpo, Rehai and Qinghai) and Longling town (Banglazhang), Yunnan Province, China. Sampling was conducted from 16 different sites including water, wet sediment, and microbial mats during August 2017, targeting especially cyanobacterial population and their isolation (Figure 1 and Table 1). Limnological features like pH, temperature (°C), and conductivity (μS/cm) were recorded using a portable pH meter equipped with a pH probe, a temperature probe, and a conductivity probe (Thermo Orion 420C-01A), respectively, on the spot. Microbial biofilms from various substrates were scrapped using sterile forceps and spatula into 50-ml Falcon tubes. Water samples were collected in pre-sterilized 1-L polyethylene bottles for chemical analysis. These samples were immediately stored on dry ice for transportation and later kept at a −80°C deep freezer in the laboratory until further analysis. Limnological parameters including concentration of cations (calcium, magnesium, potassium, ammonium, sodium, and lithium) and anions (nitrite, nitrate, phosphate, sulfate, bromide, chloride, and fluoride) were analyzed using Dionex ICS-5000 + ion-exchange chromatography system equipped with Dionex IonPac CS12A and Dionex IonPac CS18 column (250 × 4 mm, 5 μm, ThermoFisher, Waltham, MA, United States), respectively.

**TABLE 1:** Description of the sampling sites, hot spring samples, and environmental parameters (Yunnan Province).

Place with sample no.	Sample code	GPS data (N/E)	Sample description	pH	Temperature (°C)	Conductivity (μS/cm)
**Tengchong town**						
Dongshan-001	Ds001	24°59′10.18″/98°31′12.76″	Green mat	9.4	43	271
Huangpo-002	Hp002	24°58′32.16″/98°32′27.64″	Blackish soil	–	–	–
Huangpo-003	Hp003	24°58′37.13″/98°32′30.91″	Green sandy mat	9.4	38	153
Rehai (Hamazui)-004	Re004	24°56′59.75″/98°26′13.09″	Green sandy mat	8.3	58	1953
Rehai (Hamazui)-005	Re005	24°56′59.75″/98°26′12.84″	Green sandy mat	7.4	60	1479
Rehai (Yanjingquan)-006	Re006	24°57′4.46″/98°26′9.78″	White sandy sediments	9.3	85	268
Rehai (Zhenzhuquan)-007	Re007	24°57′3.53″/98°26′10.1″	Grey sandy sediments	4.0	85	416
Rehai (Dagunguo)-008	Re008	24°57′12.2″/98°26′16.62″	Green mat	7.6	90	4
Rehai (Dagunguo)-009	Re009	24°57′12.49″/98°26′16.98″	Green and yellow mat	–	–	–
Qingshui-010	Qs010	24°56′44.56″/98°25′52.97″	Green soil mat	6.4	42	1391
**Longling town**						
Banglazhang-011	Blz011	24°39′18.5″/98°40′4.19″	Green mat	6.1	60	303
Banglazhang-012	Blz012	24°39′18.43″/98°40′4.19″	Green mat	8.2	88	981
Banglazhang-013	Blz013	24°39′18.36″/98°40′4.22″	Green mat	7.6	72	993
Banglazhang-014	Blz014	24°39′18.25″/98°40′4.26″	Green mat	7.6	57	748
Banglazhang-015	Blz015	24°39′18.36″/98°40′4.84″	Green soil mat	7.8	78	1021
Banglazhang-016	Blz016	24°39′17.6″/98°40′4.58″	Green mat on egg shell	7.6	87	846

### 16S rRNA Amplicon Sequencing and Data Analysis

The next-generation sequencing library preparations and Illumina MiSeq sequencing were conducted at GENEWIZ, Inc. (Suzhou, China). DNA samples were quantified using a Qubit 2.0 Fluorometer (Invitrogen, Carlsbad, CA, United States). DNA (30**–**50 ng) was used to generate amplicons using a MetaVx™ Library Preparation kit (GENEWIZ, Inc., South Plainfield, NJ, United States). Primers targeting V3–V4 hypervariable regions of the 16S rRNA gene were proved to be able to capture the bacterial diversity in various environments (Klindworth et al., 2013). Thus, these regions were selected to generate amplicons and follow taxonomy analysis. These regions were amplified using primer pairs V3V4-F (CCTACGGRRBGCASCAGKVRVGAAT) and V3V4-R (GGACTACNVGGGTWTCTAATCC). First-round polymerase chain reaction (PCR) products were used as templates for second-round amplicon enrichment PCR. At the same time, indexed adapters were added to the ends of the 16S rRNA amplicons to generate indexed libraries ready for downstream NGS sequencing on Illumina Miseq. DNA libraries were validated by Agilent 2100 Bioanalyzer (Agilent Technologies, Palo Alto, CA, United States), and quantified by Qubit 2.0 Fluorometer. DNA libraries were multiplexed and loaded on an Illumina MiSeq instrument according to the manufacturer’s instructions (Illumina, San Diego, CA, United States). Sequencing was performed using a 2 × 300 paired-end (PE) configuration; image analysis and base calling were conducted by the MiSeq Control Software (MCS) embedded in the MiSeq instrument. The QIIME data analysis package was used for 16S rRNA data analysis. The forward and reverse reads were joined and assigned to samples based on barcode and truncated by cutting off the barcode and primer sequences. Quality filtering on joined sequences was performed and sequences with length < 200 bp, ambiguous bases, and mean quality score ≥ 20 were discarded. Then, the sequences were compared with the reference database (RDP Gold database) using the UCHIME algorithm to detect chimeric sequences, and then the chimeric sequences were removed. The effective sequences were used in the final analysis. Sequences were grouped into operational taxonomic units (OTUs) using the clustering program VSEARCH (1.9.6) against the Silva 119 database pre-clustered at 97% sequence identity. The Ribosomal Database Program (RDP) classifier was used to assign taxonomic category to all OTUs at a confidence threshold of 0.8. The RDP classifier uses the Silva 123 database, which has taxonomic categories predicted to the species level.

### Isolation and Culturing of Cyanobacteria Coupled With Temperature Stress Experiments

The biological mats were washed thoroughly by double-distilled water to remove inorganic debris, followed by homogenization by a glass homogenizer (1 ml) and then inoculated into Petri plates containing solidified BG11 medium (Rippka et al., 1979) at a slightly higher temperature of up to 40 ± 2°C under 30 μmol photon m^–2^ s^–1^ continuous light intensity in a temperature-controlled culture room. Axenic cultures of cyanobacteria were obtained by repeated sub-culturing on Petri plates.

To verify the thermotolerant features of these isolated cyanobacterial species, they were incubated in 10-ml test tubes at high temperature (50°C) with initial OD_730_ ∼0.06 under a continuous light intensity of 30 μmol photon m^–2^ s^–1^ without shaking.

### Microscopy

Morphological features of the cyanobacteria were studied extensively under an Olympus CX31 microscope equipped with an Olympus DP-72 digital camera (Olympus, Tokyo, Japan) and software DP2-BSW (version 2.2) at 20 ms exposure time. The enumeration of phenotypic features was later considered for the identification of the taxa.

### DNA Extraction and Polymerase Chain Reaction Amplification of 16S rRNA Gene of Isolated Cyanobacteria

Genomic DNA of all the isolates was extracted by using HiPurA™ plant genomic DNA Miniprep Purification Spin Kit. 16S rRNA gene from the isolated DNA was amplified using cyanobacteria-specific primers CYA106F and CYA781R (Nübel et al., 1997). PCR amplification consisted of an initial denaturation at 95°C for 5 min, 32 cycles of denaturation at 95°C for 30 s, annealing at 55°C for 1 min, and extension at 72°C for 1 min, and a final extension at 72°C for 10 min. Individual reagents and their concentrations were as follows: 1 × PCR buffer with 1.5 mM MgCl_2_, dNTPs (100 μM each), 0.25 μM each primer, 2.5 U of DNA polymerase (Ex-Taq) (TaKaRa, Japan), and ∼50 ng of total DNA. PCR products were purified using an E.Z.N.A. Gel Extraction Kit (Omega Bio-Tek, United States) according to the manufacturer’s instructions. The obtained sequences of 16S rRNA gene were sequenced and the ambiguities were removed by visual editing. The sequences were compared with the NCBI GenBank database using the BLASTn^1^ and sequences of uncultured organisms were excluded.

### Nucleotide Sequence Data Deposition

The paired-end nucleotide sequences have been submitted in NCBI SRA database with the accession IDs SRR11796781–SRR11796799 and SAMN14918520–SAMN14918537 for BioSample accessions under the BioProject PRJNA628565. Sequences of 16S rRNA genes obtained during the present study were deposited in GenBank with accession numbers (MK625304–MK625325).

### Phylogenetic Analyses

All OTUs belonging to cyanobacteria and 16S rRNA gene sequence of cyanobacterial isolates were compared with the NCBI (National Center for Biotechnology Information) database using BLAST. An initial BLAST search of the NCBI GenBank database provided candidate sequences for comparing relationships of the cyanobacterial isolates with previously characterized cyanobacterial species. Different sets of sequences were combined together to construct 3 types of phylogenetic trees in the present study, which are as follows: (1) the sequences of major OTUs of cyanobacteria/bacteria of corresponding sampling spots and 16S rRNA gene sequence of cyanobacterial isolates; (2) the sequences of all reported OTUs of cyanobacteria and 16S rRNA gene sequence of cyanobacterial isolates; and (3) the 16S rRNA gene sequences of other hot spring cyanobacteria reported worldwide and 16S rRNA gene sequence of cyanobacterial isolates of the present study. Multiple alignments were created with reference to the selected GenBank sequences and Neighbor-joining phylogenetic trees were constructed from dissimilar distance and pairwise comparisons with the Jukes–Cantor distance model using the MEGA (molecular evolutionary genetics analysis) program, version 5 (Tamura et al., 2011). Bootstrap replications of 1,000 were assessed.

## Results

### Microbial Mat, Physicochemical Parameters, and Mineral Composition of Hot Spring Water

Microbial mats in the sampled thermal springs were both submerged and floating on the surface (Figure 1). Sometimes they appear as gelatinous films blanketed over sandy bottoms of a few hot springs. The textures of the mats were thin slimy gelatinous films to thick leathery mats with color ranging from bluish to yellowish green. The hot springs displayed a wide range of limnological characteristics, i.e., moderate to high temperature (38–90°C), acidic to slightly alkaline pH (4.0–9.4), and a variable conductivity (4–1,953 μS/cm), though the springs are in close proximity (Table 1). Based on the water temperature, the sampling sites were segregated into four groups representing different temperature gradients, i.e., Groups 1 (30–50°C), 2 (50–70°C), 3 (70–80°C), and 4 (80–90°C). Thermal springs Ds001, Hp003, and Qs010 (Group 1) were marked with the lowest temperature range (30–50°C), while the highest temperature range (80–90°C) was documented for the springs Re006, Re007, Re008, Blz012, and Blz016 (Group 4). Springs with temperature range 50–70°C were listed for Re004, Re005, Blz011, and Blz014 (Group 2). Only two springs Blz013 and Blz015 (Group 3) had a temperature range 70–80°C.

A comparative ionic composition and conductivity of water samples are presented in supporting information (Supplementary Figure 1). The abundance of sodium ion (Na^+^), among the cations in all the water samples, was distinctly observed followed by the concentration of potassium (K^+^). Similarly, chloride and sulfate were the dominant anions. The variation in ionic concentrations and conductivity in Longling town hot springs was negligible, but significant in the hot springs of Tengchong town. Total ion analysis (cations and anions) showed its richness in water sample of Re008 and minimum in Hp003. It was also revealed that the conductivity was directly proportional to the concentration of ions present in water samples. However, the derivation of relatedness was not made for the sample Re008, due to insufficient water sampling.

### Overview of Meta-16S rRNA Gene Sequencing Data (Total Microorganisms)

A total of 584,226 reads (1787 OTUs) were identified from these samples. Operational taxonomic unit Venn or Petal diagram was also generated to illustrate the distribution of OTUs among these four groups (Supplementary Figure 2). (1) Group 1/30–50°C (1,186 OTUs): Ds001, Hp003, and Qs010; (2) Group 2/50–70°C (761 OTUs): Re004, Re005, Blz011, and Blz014; (3) Group 3/70–80°C (322 OTUs): Blz013 and Blz015, and (4) Group 4/80–90°C (713 OTUs): Re006, Re007, Re008, Blz012, and Blz016. The OTUs restricted to any individual group were 833, 103, 15, and 60 for Group 1, Group 2, Group 3, and Group 4, respectively. It also showed the information on the distribution of shared OTUs across these four groups, which were 260, 80, 253, 261, 541, and 268 between Groups 1 and 2, Groups 1 and 3, Groups 1 and 4, Groups 2 and 3, Groups 2 and 4, and Groups 3 and 4, respectively. Larger proportions of OTUs (179) were shared between Groups 2 and 4. There was relatively lower overlap between Groups 1 and 3. Common OTUs shared by all the groups was 58. Chao1 was used to compare species richness between different groups (Supplementary Figure 3), which supported that OTU estimate was significantly higher in Group 1, i.e., the lowest temperature gradient in all gradients.

Thirty major OTUs were represented as clustering heat maps (Supplementary Figure 4), which showed the color of different shades based on the relative abundance of each OTU. Among these, OTUs 1, 2, 5, 7, 12, and 21 belong to cyanobacteria, where OTUs 2 and 5 were related to morphological subsection III. Microbial community composition based on pyrosequencing of 16S rRNA gene (Supplementary Figure 5) revealed Cyanobacteria and Proteobacteria representing a larger portion of bacterial community in the hot spring mats of Yunnan Province. Other most common bacterial genera were *Firmicutes*, *Chloroflexi*, *Bacteroidetes*, *Acidobacteria*, and *Deinococcus-thermus*. Abundance of cyanobacteria was reported in all the sampling sites except RH007, which was rich with Proteobacteria (Supplementary Figure 5A). Furthermore, it can also be inferred that an extremely high temperature of 85°C and the acidic water (pH 4.0) of RH007 favored the growth of *Proteobacteria*, *Bacteroidetes*, and *Actinobacteria*, but inhibited the growth of cyanobacteria. Another site with the hottest (85°C) but alkaline water (pH 9.3), i.e., RH006, supported the growth of the members of phylum *Thermodesulfobacteria* and *Aquificae* rather than Cyanobacteria and Proteobacteria. Our result showed that the bacterial phylum Cyanobacteria and Proteobacteria were dominant in all the studied hot springs. The dominating organism in microbial mat depended on the temperature, pH, and geochemistry of sampling sites (Hou et al., 2013). Species richness of cyanobacteria was higher in the springs HP003, RH009, BLZ014, and BLZ015 than any other spots (Supplementary Figure 5A). The lowest number of OTUs was observed in Group 3, i.e., temperature range of 70–80°C, but interestingly with the highest proportion belonging to cyanobacteria (Supplementary Figure 5B).

### Overview of Meta-16S rRNA Gene Sequencing Data (Cyanobacteria)

**FIGURE 2:**
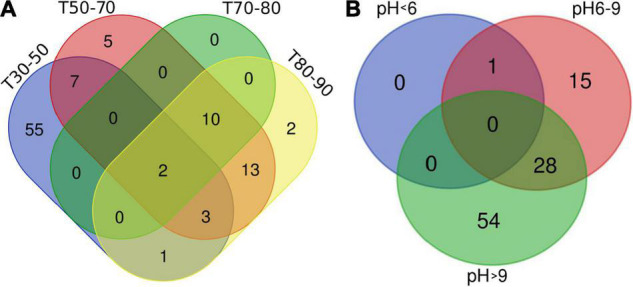
OTU Venn diagram illustrating distribution of cyanobacterial OTUs among the four groups of temperature range **(A)** and three groups of pH range **(B)**. T indicates the temperatures of the water samples or the hot spring water temperatures of other sample types. T30–50 (Group 1: 30–50°C), T50–70 (Group 2: 50–70°C), T70–80 (Group 3: 70–80°C), and T80–90 (Group 4: 80–90°C). The Venn diagram was generated using the OmicStudio tools (https://www.omicstudio.cn/tool).

**FIGURE 3:**
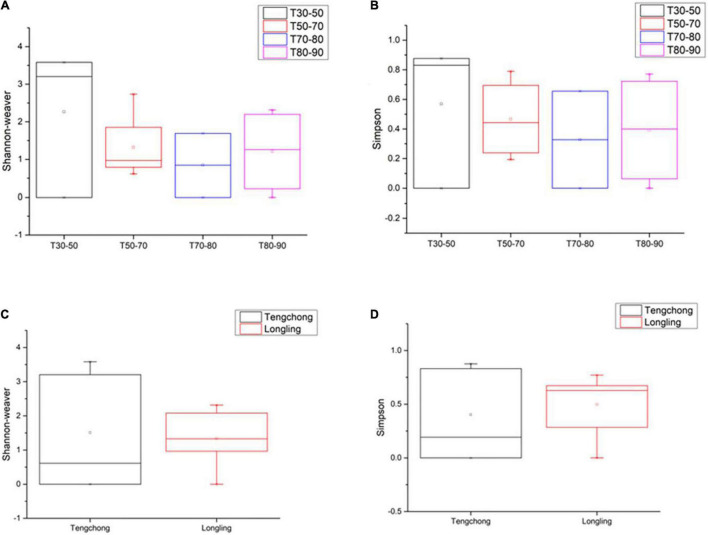
The Shannon–Weaver and Simpson indices of cyanobacterial OTUs in thermal springs based on temperature range **(A,B)** and location **(C,D).** The Box plot was generated using the Origin tools. Median values and interquartile ranges have been indicated in the plots. T indicates the temperatures of the water samples or the hot spring water temperatures of other sample types. T30–50 (Group 1: 30–50°C), T50–70 (Group 2: 50–70°C), T70–80 (Group 3: 70–80°C), and T80–90 (Group 4: 80–90°C).

This study on the microbial composition in these hot springs was intended to explore cyanobacterial diversity in more detail rather than dealing with genera of other microorganisms precisely. Out of the total OTUs, 100 OTUs belonging to cyanobacteria were identified. Ninety-four and 36 cyanobacterial OTUs were found in Tengchong and Longling town, respectively. The OTU Venn diagram was illustrated based on the distribution of cyanobacterial OTUs among the four groups of temperature range and three groups of pH range (Figure 2). It is quite clear that there were diversified cyanobacteria in hot springs with temperature 30–50°C (Figure 2A) and pH > 9 (Figure 2B). This was also supported by the Shannon–Weaver (Figure 3A) and Simpson index (Figure 3B) analysis. When grouped by region, the results show that the diversity of cyanobacteria in Tengchong has changed greatly, while the diversity in Longling town is relatively small (Figures 3C,D).

**FIGURE 4:**
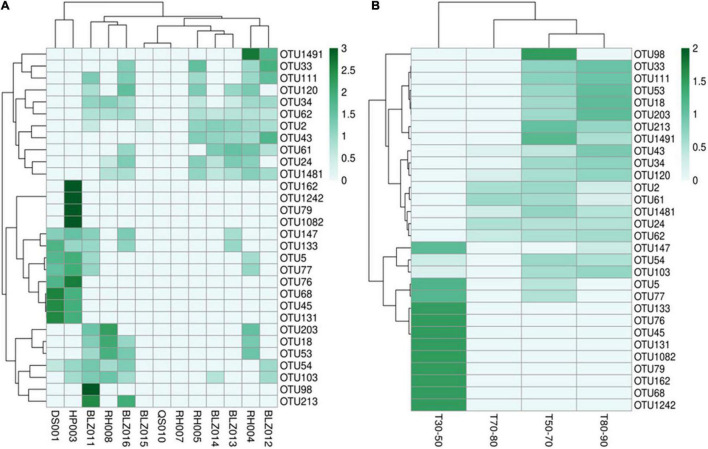
The cyanobacterial OTU abundance clustering heat map. The rows and columns represent the OTU IDs and the sampling sites **(A)** temperature groups **(B)**, respectively. The OTU clustering tree is in the left. The value of each square color of the middle heat map corresponds to the relative abundance of each row of OTU. The heat map was generated using the OmicStudio tools (https://www.omicstudio.cn/tool).

According to the abundance of cyanobacteria OTUs at each sample point, 30 OTU species with higher abundance were selected to make a heat map (Figure 4). The abundance of different cyanobacteria in each sampling point (Figure 4A) as well as in different temperature ranges was depicted (Figure 4B), which showed that the types of cyanobacteria were quite different. In particular, in the T30–50 group, the cyanobacteria were significantly different from the other three groups, and the abundance of cyanobacteria was higher. The other three groups of cyanobacteria had relatively similar types and lower abundance, which may be due to high water temperature and insufficient nutrients in the water.

**FIGURE 5:**
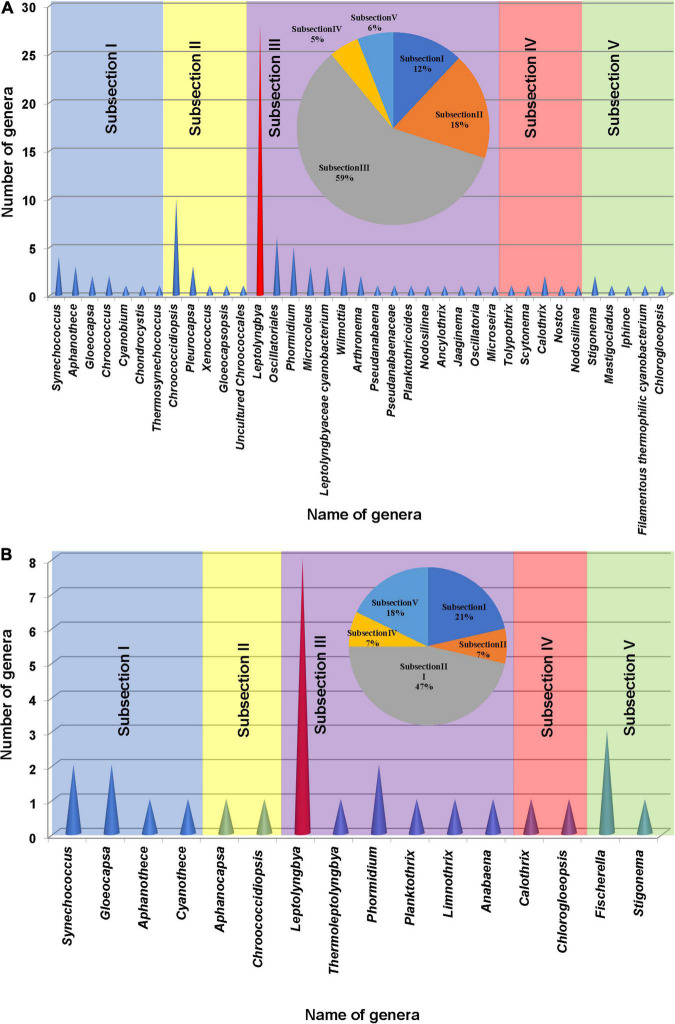
Cyanobacterial genera found based on the 16S rRNA amplicon sequencing analysis **(A)** and microscopic observation **(B)** of the samples collected from different studied sites of hot springs/hot water source of China. A pie chart was included in each histogram to display the percentage of cyanobacterial strains based on morphological subsections.

The cyanobacterial OTUs found in the present study were assigned to 34 genera and 3 cyanobacterial groups (*Synechococcus*, *Gloeocapsa*, *Aphanothece*, *Chroococcus*, *Cyanobium*, *Chondrocystis*, *Thermosynechococcus*, *Chroococcidiopsis*, *Pleurocapsa*, *Xenococcus*, *Gloeocapsopsis*, Uncultured Chroococcales, *Leptolyngbya*, *Oscillatoriales*, *Phormidium*, *Microcoleus*, Leptolyngbyaceae cyanobacterium, *Wilmottia*, *Arthronema*, *Pseudanabaena*, *Pseudanabaenaceae*, *Planktothricoides*, *Nodosilinea*, *Ancylothrix*, *Jaaginema*, *Oscillatoria*, *Microseira*, *Tolypothrix*, *Scytonema*, *Calothrix*, *Nostoc*, *Nodosilinea*, *Chlorogloeopsis*, *Stigonema*, *Mastigocladus*, *Iphinoe*, and filamentous thermophilic cyanobacterium) approximately. More than 59% of total morphological subsection was represented by subsection III (59% of total subsections), especially the members of the genus *Leptolyngbya*. The second most abundant cyanobacterial group belonged to subsection II (18%) followed by subsections I (12%), V (6%), and IV (5%) (Figure 5A).

### Phenotypic Characterization and Cyanobacterial Community Compositions

**FIGURE 6:**
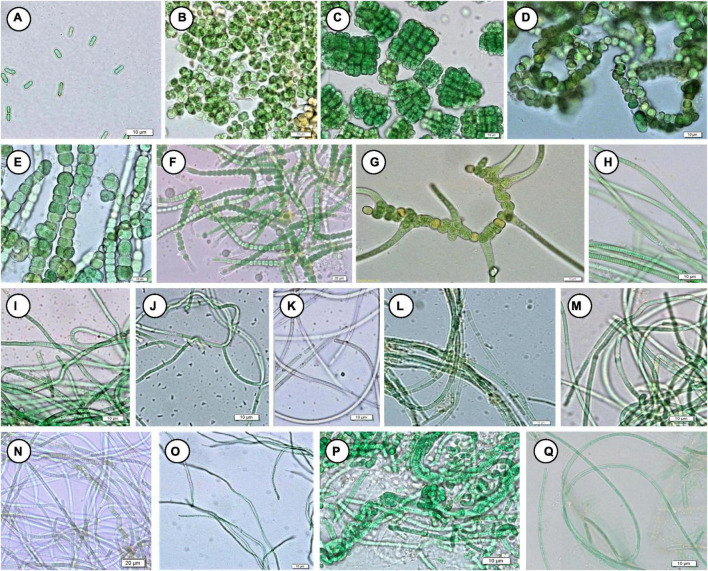
Mat-forming cyanobacteria identified in this study. **(A)** *Synechococcus* cf. *nidulans*, **(B)** *Gloeocapsa gelatinosa*, **(C)** *Chlorogloeopsis fritschii*, **(D)** *Anabaena* sp. NK1-14, **(E)** *Fischerella* sp. NK 1-16, **(F)** *Fischerella* sp. NK 1-20, **(G)** *Fischerella thermalis*, **(H)** *Planktothrix cryptovaginata*, **(I)** *Leptolyngbya copelandii*, **(J)** *L. dangeardii*, **(K)** *L. faveolarum*, **(L)** *Leptolyngbya* sp. NK 1-10, **(M)** *Leptolyngbya* sp. NK 1-23, **(N)** *Leptolyngbya* sp. NK 1-12, **(O)** *Thermoleptolyngbya oregonensis*, **(P)** *Leptolyngbya thermobia*, **(Q)** *L. valderiana*.

Floristic assessment of the mat-forming cyanobacteria and other associated algae through microscopy identified altogether 39 taxa from these thermal habitats of Yunnan Province (Supplementary Table 1). As stated, the thermal springs of Yunnan provide a wide range of habitats with diverse physicochemical parameters. The cyanobacteria in these extreme habitats thus get ample opportunity to adapt various environmental circumstances. To understand this, we tried to index the ecological specifications of the cyanobacterial species distributed in these thermal habitats. Cyanobacteria reported from Yunnan hot springs were categorized to adapt to various groups, i.e., moderate acidophilic, moderate alkaliphilic, mesothermophilic, thermophilic, and hyperthermophilic, depending on the pH and temperature of the hot springs from where they have reported/isolated (Table 2). Cyanobacteria were identified through extensive phenotypic characterizations (Figure 6 and Supplementary Figure 6). They displayed similar diversities at both species and generic levels. *Leptolyngbya* was the most diverse genus (8 taxa), followed by *Fischerella* (3 taxa). For more clarity in understanding the diversity, the taxa were grouped into different sub-sections to estimate their percentage of contribution to the biome (Figure 5B). It was revealed that the cyanobacterial species belonging to subsection III (47% of total cyanobacterial subsection diversity) were the major contributor in mat formation, which included taxa of *Leptolyngbya*, *Thermoleptolyngbya*, *Phormidium*, and *Planktothrix*, which were observed in 10 sampling sites out of the 16. *Leptolyngbya* dominated the hot springs of DS001, HP003–HP006, BLZ011, BLZ013, and BLZ014, whereas *Thermoleptolyngbya* dominated in HP006, HP007, and BLZ011. Likewise, *Fischerella* colonized the mats in QS010, BLZ012, and BLZ016. *Synechococcus*, *Gloeocapsa*, *Chlorogloeopsis*, and *Anabaena* were considered as the other associated mat-forming cyanobacterial species. It is noteworthy to mention that the sites with temperature > 80°C had only a few traces of cyanobacterial populations that were detected after extensive microscopy of the natural samples.

**TABLE 2:** Nature of reported cyanobacteria from Yunnan hot springs based on the range of pH and temperature of sampling sites.

Sl. No.	Cyanobacterial taxa	Location	Nature
1	*Synechococcus* cf. *nidulans*	Hp003	Moderate-alkaliphilic; mesothermophilic
2	*Synechococcus elongatus*	Blz016	Moderate-alkaliphilic; hyperthermophilic
3	*Gloeocapsa gelatinosa*	Ds001, Hp003	Moderate-alkaliphilic; mesothermophilicto thermophilic
4	*Gloeocapsa sanguina*	Hp003	Moderate-alkaliphilic; mesothrmophilic
5	*Cyanothece* sp.	Blz013, Blz016	Moderate-alkaliphilic; hyperthermophilic
6	*Aphanothece microscopica*	Hp003	Moderate-alkaliphilic; mesothermophilic
7	*Aphanocapsa thermalis*	Ds001, Hp003	Moderate-alkaliphilic; thermophilic
8	*Chroococcidiopsis thermalis*	Hp003	Moderate-alkaliphilic; mesothermophilic
9	*Leptolyngbya copelandii*	Re004, Re005	Moderate-alkaliphilic; thermophilic
10	*Leptolyngbya dangeardii*	Blz013	Moderate-alkaliphilic; hyperthermophilic
11	*Leptolyngbya faveolarum*	Ds001, Blz011	Moderate-acidophilic to moderate-alkaliphilic; thermophilic to hyperthermophilic
12	*Leptolyngbya thermobia*	Re006, Blz014	Moderate-alkaliphilic; thermophilic to hyperthermophilic
13	*Leptolyngbya valderiana*	Blz011	Moderate-acidophilic; hyperthermophilic
14	*Leptolyngbya* sp. NK 1-10	Hp003	Moderate-alkaliphilic; mesothermophilic
15	*Leptolyngbya* sp. NK 1-12	Hp003	Moderate-alkaliphilic; mesothermophilic
16	*Leptolyngbya* sp. NK 1-23	Hp003	Moderate-alkaliphilic; mesothermophilic
17	*Thermoleptolyngbya oregonensis*	Re007, Re008, Blz011	Moderate-acidophilic to moderate-alkaliphilic; thermophilic to hyperthermophilic
18	*Phormidium ambiguum*	Ds001, Hp003, Re004, Blz012	Moderate-alkaliphilic; thermophilic to hyperthermophilic
19	*Phormidium terebriforme*	Blz013, Blz014	Moderate-alkaliphilic; thermophilic to hyperthermophilic
20	*Planktothrix cryptovaginata*	Hp003	Moderate-alkaliphilic; mesothrmophilic
21	*Limnothrix* sp.	Re005	Moderate-alkaliphilic; thermophilic
22	*Anabaena* sp. NK1-14	Re004	Moderate-alkaliphilic; thermophilic
23	*Calothrix* sp.	Hp003	Moderate-alkaliphilic; mesothermophilic
24	*Chlorogloeopsis fritschii*	Blz016	Moderate-alkaliphilic; hyperthermophilic
25	*Fischerella thermalis*	Ds001	Moderate-alkaliphilic; thermophilic
26	*Fischerella* sp. NK 1-16	Qs010	Moderate-acidophilic; thermophilic
27	*Fischerella* sp. NK 1-20	Blz012	Moderate-alkaliphilic; hyperthermophilic
28	*Fischerella* sp. NK 1-21	Blz016	Moderate-alkaliphilic; hyperthermophilic

*Mesothermophilic (20 to 40°C); thermophilic (40 to 60°C); hyperthermophilic (≥ 60°C); moderate acidophilic (pH 3–6.9); moderate alkaliphilic (pH 7.1–10).*

### Comparison Between 16S rRNA Amplicon Sequencing and Phenotypic Observations

Operational taxonomic units (OTUs) belonging to genera *Cyanothece*, *Aphanocapsa*, *Thermoleptolyngbya*, *Planktothrix*, *Limnothrix*, *Anabaena*, and *Fischerella* were not identified in 16S rRNA sequencing, which may be due to insufficient samples/DNA quantity for high-throughput sequencing study as well as insufficient data in GenBank. The common genera in both 16S rRNA sequencing and phenotypic analysis were *Synechococcus*, *Gloeocapsa*, *Aphanothece*, *Chroococcidiopsis*, *Leptolyngbya*, *Phormidium*, *Calothrix*, and *Chlorogloeopsis*. Other cyanobacterial taxa reported in 16S rRNA sequencing analysis were not identified through microscopic observations because of their rarity in occurrence. We believe that the phenotypic characterization of any particular group of microorganisms, especially cyanobacteria, will impart more authenticity along with the 16S rRNA sequencing analysis in understanding the infrastructure of any microbial ecosystem. Further, the available genetic resource databases lack thorough taxonomic information including the identity of a species. Thus, we have adopted both methods together to reveal true cyanobacterial diversity in the hot springs of Yunnan Province, which was represented by a total of 45 cyanobacterial genera. The 16S rRNA sequencing analysis depicted the whole cyanobacterial diversity of the habitats with 100 different types of taxa, but their population and pattern of colonization were deeply elaborated after the phenotypic characterization, where the dominant mat-forming and associated taxa were segregated.

### Isolation of Hot Spring Cyanobacteria and Verification of Their Thermotolerances

A total of 19 cyanobacterial strains belonging to 10 species and 6 genera were isolated to axenic cultures. These isolates will create the chance to understand the physiology of heat-tolerant cyanobacteria, screen for value-added products, and develop them as model microorganisms. They were closely observed for morphological alterations during their various growth phases, to facilitate more authentic identification. Their closest relatives in database (percentage similarity) and taxonomic assignment are presented in Table 3. Most of the isolates were identified up to species level except *Leptolyngbya* sp. NK 1-10, *Leptolyngbya* sp. NK 1-12, *Anabaena* sp. NK1-14, *Fischerella* sp. NK 1-16, *Fischerella* sp. NK 1-20, *Fischerella* sp. NK 1-21, and *Leptolyngbya* sp. NK 1-23. All these taxa were not identified up to species level, which could open the scope for identifying them as novel taxa with biotechnological potential in the near future.

**TABLE 3:** List of identified cyanobacterial isolates from representative hot springs of Yunnan Province, China.

Sl No.	Code	Location	Closest relatives in NCBI database (% similarity)	Morphology identification and taxonomic assignment	Accession No.
1	NK1-7	Dongshan-001	Uncultured Oscillatoriales cyanobacterium (KJ611462), 99.84%	*Leptolyngbya faveolarum*	MK625308
2	NK1-10	Huangpo-003	*Leptolyngbya* sp. (KF746954), 100%	*Leptolyngbya* sp.	MK625309
3	NK1-11	Huangpo-003	*Planktothrix pseudagardhii* (JQ894510), 95.10%	*Planktothrix cryptovaginata*	MK625310
4	NK1-12	Huangpo-003	Oscillatoriales cyanobacterium (KU557673), 99.83%	*Leptolyngbya* sp.	MK625311
5	NK1-13	Rehai (Hamazui)-004	*Leptolyngbya* sp. (MG753795), 97.21%	*Leptolyngbya copelandii*	MK625312
6	NK1-14	Rehai (Hamazui)-004	*Anabaena* sp. (HM235817), 98%	*Anabaena* sp.	ND
7	NK1-15	Rehai (Dagunguo)-008	*Leptolyngbya* sp. (AP017367), 99.83%	*Thermoleptolyngbya oregonensis*	MK625313
8	NK1-16	Qingshui-010	*Hapalosiphon* sp. (LC325255), 98.21%	*Fischerella* sp.	MK625314
9	NK1-18	Banglazhang-011	*Leptolyngbya* sp. (KT899570), 97.63%	*Leptolyngbya valderiana*	MK625315
10	NK1-19	Banglazhang-011	Uncultured Oscillatoriales cyanobacterium (KJ611462), 99.67%	*Leptolyngbya faveolarum*	MK625316
11	NK1-20	Banglazhang-012	*Fischerella* sp. (MG015899), 100%	*Fischerella* sp.	MK625317
12	NK1-21	Banglazhang-016	*Fischerella* sp. (MG015899), 100%	*Fischerella* sp.	MK625318
13	NK1-22	Banglazhang-011	*Leptolyngbya* sp. (AP017367), 99.67%	*Thermoleptolyngbya oregonensis*	MK625319
14	NK1-23	Huangpo-003	*Leptolyngbya* sp. (MK487619), 97.82%	*Leptolyngbya* sp.	MK625320
15	NK1-24	Banglazhang-016	*Chlorogloeopsis fritschii* (KT807481), 100%	*Chlorogloeopsis fritschii*	MK625321
16	NK1-25	Rehai (Yanjingquan)-006	*Leptolyngbya boryana* (MF629803), 100%	*Leptolyngbya thermobia*	MK625322
17	NK1-26	Rehai (Zhenzhuquan)-007	*Leptolyngbya* sp. (AP017367), 99.83%	*Thermoleptolyngbya oregonensis*	MK625323
18	NK1-27	Banglazhang-013	*Leptolyngbya* sp. (MG822743), 99.83%	*Leptolyngbya dangeardii*	MK625324
19	NK1-28	Banglazhang-014	*Leptolyngbya copelandii* (KM376984), 99.83%	*Leptolyngbya thermobia*	MK625325

**FIGURE 7:**
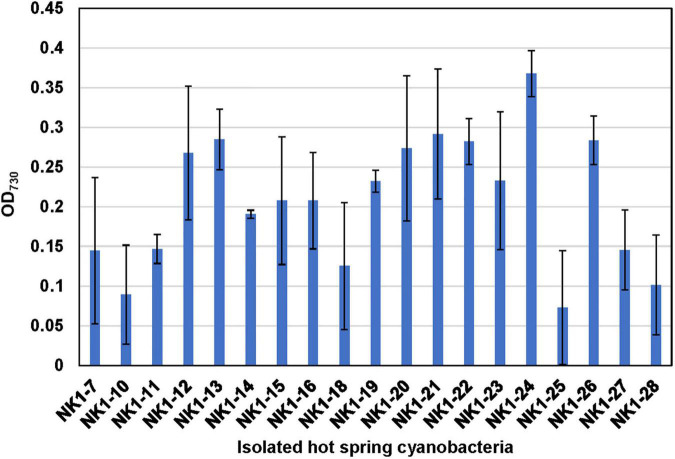
Verification of the thermotolerant feature of the hot spring cyanobacterial isolates. Note that the isolated cyanobacterial species was grown statically in 10-ml test tubes at 50°C under 30 μmol photon m^–2^ s^–1^ light intensity for 10 days.

With the aim of confirming the thermotolerant feature of these isolated strains and evaluating their survival capacity at higher temperature, they were incubated at OD_730_ ∼0.06 in test tubes and cultivated statically at 50°C under 30 μmol photon m^–2^ s^–1^ light intensity for 10 days. Compared to their initial OD_730_, most of the cultures, except for NK1-10 and NK1-25, exhibited increased cell densities under these rigorous laboratory culture conditions (Figure 7). The results indicated the survivability of our isolates even at very low cell density under high temperature and further highlighted the significance of hot spring cyanobacterial strain mining using integrative approach.

### Phylogenetic Analysis of Cyanobacteria

Phylogenetic relationships among the 16S rRNA gene sequences of isolated cyanobacterial species were analyzed along with the OTUs reported from our next-generation sequencing as well as other 16S rRNA gene sequences of related taxa. The OTUs obtained through next-generation sequencing were analyzed by NCBI BLAST search (one by one) and classified into different subsections (Supplementary Table 2). A total of 100 cyanobacterial OTUs were confirmed, which mainly belonged to subsection III and *Leptolyngbya* genera. Major OTUs of corresponding sampling spots and 16S rRNA gene sequences of isolated cyanobacteria species from the hot springs of Yunnan Province were compared, which showed that isolated cyanobacterial species belonged to same subsection as major cyanobacterial OTUs of the corresponding sampling site (Supplementary Table 3).

**FIGURE 8:**
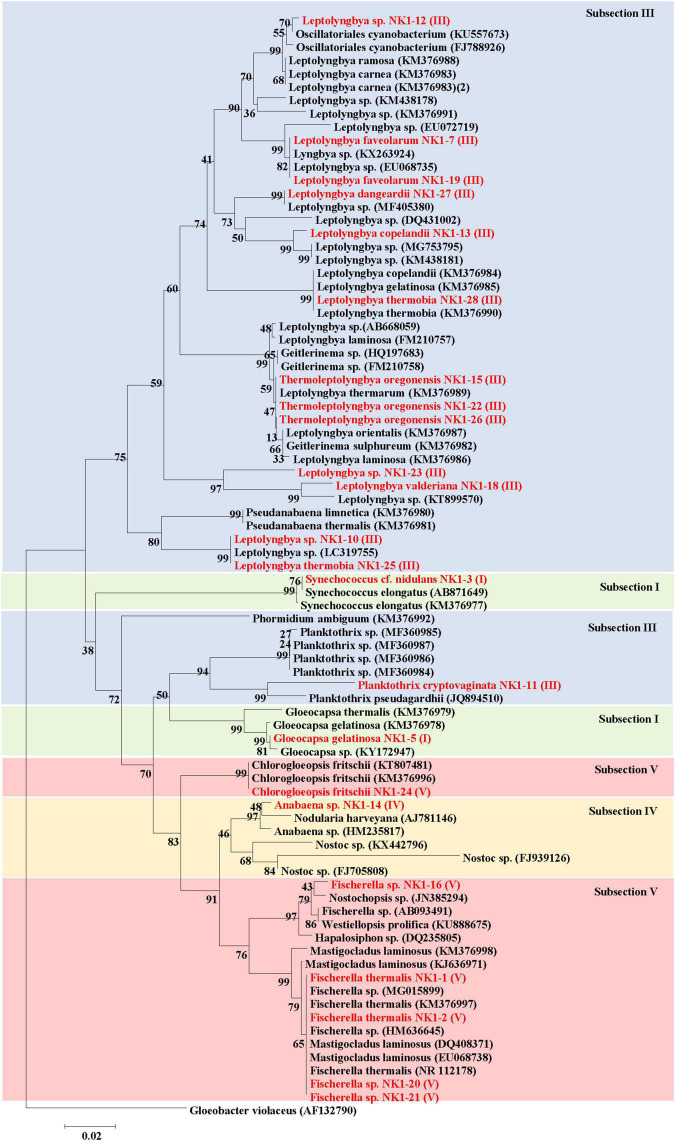
Phylogenetic relationships (Neighbor-Joining) between the isolated cyanobacteria species of hot springs of Yunnan Province, China and other closest relative hot spring cyanobacteria based on 16S rRNA gene sequences. Isolated cyanobacteria were indicated in red color. The number near the node represents the bootstrap value. Subsection was mentioned in brackets. *Gloeobacter violaceus* PCC 7421 (AF132790) was used as an out-group.

Different dendrograms of isolated cyanobacterial species were constructed using the following information: (1) major OTUs of cyanobacteria and microorganisms reported from 16S rRNA sequencing analysis (Supplementary Figure 7); (2) all OTUs of cyanobacteria reported in 16S rRNA sequencing (Supplementary Figure 8); and (3) already reported cyanobacteria isolated from hot springs (Figure 8).

The sequences of major OTUs of corresponding sampling spots and 16S rRNA gene sequences of isolated cyanobacteria species from the hot springs of Yunnan Province were combined and Neighbor-joining phylogenetic trees were constructed (Supplementary Figure 7). The result showed that OTUs from 16S rRNA sequencing and isolated cyanobacterial species, belonging to the same subsection, clustered together. A major clade belonged to subsection III followed by subsection V. The OTUs 18, 33, 54, and 722 belonged to subsection III and clustered with different species of *Leptolyngbya*, *Thermoleptolyngbya*, and *Planktothrix* (isolated organism of the present study). *Chlorogloeopsis fritschii* NK1-24 was clustered with OTU1619, which showed the BLAST search similarity with *C. fritschii* (96%). Our isolated *Fischerella* sp. NK1-16, 20, and 21 clustered together with OTU298 under subsection V. OTUs 2 and 5 were the dominating cyanobacterial OTUs, which were classified as *Uncultured Oscillatoriales* and *Phormidium autumnale* based on the NCBI BLAST search, respectively. They clustered together and separated from the main subsection III clade. Other major OTUs belonging to bacterial taxa (OTU3, OTU4, OTU7, OTU8, OTU20, and OTU27) made a separate clade in the dendrogram.

Phylogenetic relationships between the isolated cyanobacterial species and all OTUs (100 OTUs) obtained through 16S rRNA sequencing of hot springs of Yunnan Province, China showed the clustering of isolated cyanobacteria in two major clades (Supplementary Figure 8). These two clades belong to subsections III, IV, and V. There were many other clades belonging to subsections I, II, and III, but showed separate clustering from our isolated organisms.

The 16S rRNA gene sequences of the isolated cyanobacteria were from BLAST in NCBI and sequences of other related taxa reported from hot springs were retrieved for the construction of a dendrogram (Figure 8). Phylogenetic analyses revealed that subsection III cyanobacteria were clustered in two clades. The first major clade of subsection III comprised different species of genera *Leptolyngbya*, Oscillatoriales cyanobacterium, *Geitlerinema*, *Thermoleptolyngbya*, and *Pseudanabaena*, while the second clade of subsection III included only *Planktothrix* and *Phormidium*. Cyanobacteria under Subsection V, i.e., *Fischerella*, *Mastigocladus*, *Hapalosiphon*, *Westiellopsis*, and *Nostochopsis*, were clustered together, while *C. fritschii* made a separate clade. Cyanobacterial members of subsection IV formed monophyletic clade including *Anabaena* and *Nostoc*. Subsection I clustered in different clades comprising mainly *Gloeocapsa*, *Nodularia*, and other hot spring cyanobacteria.

## Discussion

Thermal habitats of China, specifically from the Tibetan plateau (Hongmei et al., 2005; Lau et al., 2009; Song et al., 2013), Yunnan Province (Xue et al., 2001; Lin et al., 2002; Xiang et al., 2003; Du et al., 2005; Song et al., 2009, 2010, 2013; Jiang et al., 2010; Hedlund et al., 2012, 2015; Pagaling et al., 2012; Hou et al., 2013; Li et al., 2015a), and Sichuan Province (Tang et al., 2018), were studied extensively for the last two decades because of their microbial diversity, taxonomy, basic physiology, cultivation, and biotechnological potential (Li et al., 2015b). While all previous studies were confined to various groups of bacteria (except cyanobacteria) and archaea, the present endeavor documented the cyanobacterial diversity in microbial mats from the hot springs of Yunnan Province, following both traditional microscopic methods as well as meta-genomic analysis of natural samples, followed by isolation of thermotolerant strains.

A wide physicochemical diversity of these springs (ambient to 97°C; pH from ≤1.8 to ≥9.3) provides a multitude of niches for extremophilic microorganisms and mainly limits them to bacteria, cyanobacteria, and archaea by restricting the other forms of life. Consistency in physicochemical properties in these hot springs can easily be observed by comparing our records with that of an earlier investigation that happened about a decade ago (Hedlund et al., 2012). This unchanged limnology provides a suitable ambience for thermotolerant species to adapt and thrive in these ecosystems. Species richness of microbes in thermal springs is dependent on temperature, pH, conductivity, and geochemistry of sampling sites (Hou et al., 2013). Sometimes, a single factor can regulate the richness of a particular species, which can be evidenced from the thermal habitats of Copahue ponds (Urbieta et al., 2014) and Garbanabra hot springs in Eritrea (Ghilamicael et al., 2017). The similar pattern was also observed for the thermal springs of Yunnan Province, where the total microbial richness was the lowest in Group 3 (70–80°C) springs, while, the highest richness was observed in Group 1 (30–50°C) springs. This supports the hypothesis by Miller et al. (2009) on the temperature regulation of the diversity of microorganisms in hot spring mats.

The hot springs of Yellowstone National Park have been used as the hot spot to explore the microbial community structure for many decades, which were dominated by Cyanobacteria and *Chloroflexi* (Ward et al., 1998, 2006; Klatt et al., 2011, 2013). The major phylotypes, including Cyanobacteria, *Proteobacteria*, *Firmicutes*, *Chloroflexi*, *Acidobacteria*, and *Deinococcus-Thermus*, have also been reported in the other hot springs at various geographical locations, i.e., China (Pagaling et al., 2012; Song et al., 2013), India (Mehetre et al., 2016; Saxena et al., 2016; Singh and Subudhi, 2016), Africa (Tekere et al., 2011; Ghilamicael et al., 2017), Malaysia (Chan et al., 2015), Kenya (Kambura et al., 2016), and Argentina (Urbieta et al., 2014). The combination of thermophilic cyanobacteria and anoxygenic photosynthetic bacteria seems to develop the photosynthetic microbial mats in diversified hot springs (Bateson et al., 1989; Ward et al., 1998).

Cyanobacteria in the hot springs of Yunnan mainly belonged to subsection III (filamentous form), especially *Leptolyngbya* as the most dominating genus. Therefore, it is believed to be the dominant and pioneer component of microbial mats in terms of biomass which governs the colonization of these mats and flow of nutrients within. Cyanobacteria need light, CO_2_, and N_2_ to fulfill their major requirements for growth, which makes them float. Hence, most of the cyanobacterial mats in hot springs of the present study were observed as floating on the surface of the water. The coccal *Synechococcus* and the filamentous heterocystous branched Cyanobacteria, like *Mastigocladus* and *Fischerella*, were the dominant mat-forming species around the globe (Castenholz, 1969; Ferris and Ward, 1997; Sompong et al., 2005; Miller et al., 2006, 2009; Ward et al., 2012). However, the *Leptolyngbya* dominated mats were also observed in several thermal springs (McGregor and Rasmussen, 2008; Moro et al., 2010; Coman et al., 2013; Mackenzie et al., 2013; Amarouche-Yala et al., 2014; Roy et al., 2015; Singh et al., 2018).

The study based on 16S rRNA amplicon sequencing and microscopic analysis together gave a clearer information on the cyanobacterial diversity. A total of 100 cyanobacterial OTUs comprising 37 genera were confirmed based on 16S rRNA gene pyrosequencing. Microscopic analysis also revealed cyanobacterial morphotypes and represented 27 cyanobacterial taxa belonging to 16 genera. The combined approach of morphological and high-throughput sequencing identified a wider cyanobacterial generic diversity (45 genera) in the hot springs of Yunnan Province, which were comparatively higher than the cyanobacterial diversity reported from the hot springs of other areas like Australia (McGregor and Rasmussen, 2008), Thailand (Sompong et al., 2005), and Bulgaria (Lukavský et al., 2011). However, 16S rRNA amplicon sequencing has revealed a much broader diversity of cyanobacteria than represented in culture, some taxa (of genera *Cyanothece*, *Aphanocapsa*, *Thermoleptolyngbya*, *Planktothrix*, *Limnothrix*, *Anabaena* and *Fischerella*), identified by microscopy, in 16S rRNA sequencing. Other cyanobacterial taxa reported in 16S rRNA amplicon sequencing were not reported in microscopic observation. It is evidenced that a cyanobacterial diversity study based on 16S rRNA amplicon sequencing alone often fails to recognize some taxa due to insufficient samples/DNA quantity as well as insufficient information in GenBank. Conversely, microscopic studies may fail to detect rare taxa due to limited resolution and a small part of microscopic samples when conducting analyses of cyanobacterial diversity of natural populations. *Synechococcus*, *Gloeocapsa*, *Aphanothece*, *Chroococcidiopsis*, *Leptolyngbya*, *Phormidium*, *Calothrix*, and *Chlorogloeopsis* were common genera in both 16S rRNA amplicon sequencing and microscopic analysis.

Almost all the cyanobacteria were moderately alkaliphilic (pH 7.1–10) except *Leptolyngbya faveolarum* and *Thermoleptolyngbya oregonensis*, which grew in moderately acidic springs (pH 4.0–6.1). *Fischerella* species generally occur in thermal springs of pH value > 5.0. In our study sites, *Fischerella* sp. NK 1-16 was found growing at a slightly acidic pH of 6.1, and the other three taxa of the genus, i.e., *Fischerella* sp. NK 1-20, *Fischerella* sp. NK 1-21, and *F. thermalis*, occurred in alkaline springs. About one-third of the cyanobacterial taxa preferred low temperature (20–40°C), specifically the members of the genera, *Gloeocapsa*, *Aphanothece*, *Chroococcidiopsis*, *Planktothrix*, and *Calothrix*. All the unidentified *Leptolyngbya* species were found to be mesothermophilic. However, *Gloeocapsa gelatinosa* was also recorded growing at a temperature slightly above 40°C in the hot springs of Dongshan in association with *Aphanocapsa thermalis* and *Fischerella thermalis*. *Synechococcus*, in general considered to be extremely thermotolerant, was reported to grow even at temperatures > 100°C around the globe. In the thermal springs of Yunnan Province, *Synechococcus cf. nidulans* was collected from Huangpo springs at 38°C and *S. elongatus* was found at 87°C in Banglazhang hot springs. *Cyanothece* species was also found to be hyperthermophilic. The most diverse genus in all these thermal springs, i.e., *Leptolyngbya*, was highly thermoadaptive, occurring in a wide temperature range of 38–85°C. The most adaptive ones were *L. faveolarum* and *L. thermobia*. Likewise, *T. oregonensis* was also found to be highly adaptive, with its distribution in a wide range of temperature (60–90°C) and pH (4.0–7.6). *Leptolyngbya* and its alied genera were also found dominating in the thermal habitats of Sichuan Province, China (Tang et al., 2018). Both the *Phormidium* species in these springs, despite preferring slightly alkaline habitats, seem to be thermoadaptive (38–88°C). *C. fritschii*, *Fischerella* sp. NK 1-20, and *Fischerella* sp. NK 1-21 were recorded from high temperatures (87–88°C) in the hot springs of Banglazhang. The springs of Rehai (Re008) have the highest chloride ion concentration and temperature (90°C) with pH 7.6, which favored the growth of only *Thermoleptolyngbya*, even on the adjacent soils. Rehai spring Re007 with a low pH (pH 4) and a high temperature (85°C) coupled with higher sulfur contents also favored the growth of only *Thermoleptolyngbya* species. It was also reported that the members of this genus inhabited the hot springs with pH 6.1 and temperature 60°C. However, in Yunnan Province, it was less distributed in other hot springs, possibly due to insufficient time for their dispersal and being outcompeted by predominant cyanobacterial strains (*Leptolyngbya*) therein. Microbiological studies on the Rehai hot springs also presented very low diversity, exclusively for *Sulfolobales* and their viruses at higher temperature (Hedlund et al., 2012). Lower microbial diversity in the hot springs of Tengchong, Yunnan Province was also concluded by Hou et al. (2013) along with non-significant correlation of diversity with geochemistry. Another important aspect that was observed in the study is that the adaptability of several cyanobacterial taxa in varied temperatures may also be inhibited by the increase in acidity of the springs. A similar pattern was also observed in the hot springs of Kenya, East Africa (Dadheech et al., 2013). However, there is no clear relation between distribution of a particular strain and the potential niche-determining parameters that explain the geographical distribution of these strains. Declined relative abundances of *Deinococcus-Thermus*, Cyanobacteria, and *Chloroflexi* were previously observed with increasing temperature in Tibetan hot springs, except for *Aquificae*, which remained constant throughout (Wang et al., 2013). The thermal springs with temperature higher than 75°C were reported for only few cyanobacterial strains in the present study. However, cyanobacterial diversity in the hot springs of Dongshan (Ds001), Huangpo (Hp003), and Rehai (Re004) with temperature range 38–58°C was the highest, which may provide optimal conditions for speciation of these strains. If pH is also considered with temperature range, it showed that hot springs Ds001 and Hp003 with pH 9.4 coupled with a moderate temperature range (38–43°C) had higher cyanbacterial report (14 taxa) than Re006 with pH 9.3 and temperature 85°C (only one species).

The occurrence of different cyanobacterial strains in these habitats was still not very clear based on environmental parameters and was likely to be caused by the combination of varied temperature, pH, and mineralogy of water and nearby soil/sediments. It is challenging to understand the community composition of diversified cyanobacterial strains of these hot springs using trait-based approaches because measured parameters might not be sufficient enough to analyze its role in shaping the community structure or important factors may go unmeasured. It was observed that temporal variation in physicochemical parameters may trigger the microbial community structure and diversity in a particular habitat (Wang et al., 2014). One study also suggests that the biotic interactions may be strong niche determinants, rather than relevant abiotic factors (nutrients, temperature, etc.), which are either unknown or cannot be precisely determined (Tromas et al., 2020). Moreover, the current findings significantly expand the knowledge of cyanobacterial distribution in diversified Yunnan hot springs. It was also observed that not all cyanobacterial species can be cultured in lab conditions. The trail for the isolation of many uncultured strains always opens the scope for research on these extreme habitats.

Hot springs are generally considered as “island-like habitats” for extremophilic organisms leading to geographical isolation (Ramette and Tiedje, 2007). Many potential and new cyanobacterial novelties were studied from China recently based on a culture-dependent approach (Tang et al., 2018). Therefore, the culture-dependent strategy was also implemented in the present study to explore culturable cyanobacterial taxa and their isolation from hot springs, in addition to a culture-independent strategy (16S rRNA amplicon sequencing and microscopic observation of natural samples). In a total of 19 cyanobacterial isolates, 6 genera were cultured, which were also characterized by 16S rRNA gene sequencing for confirmation and showed the congruency with corresponding morphological features. However, *Leptolyngbya* sp. NK 1-10, *Leptolyngbya* sp. NK 1-12, *Fischerella* sp. NK 1-16, *Fischerella* sp. NK 1-20, *Fischerella* sp. NK 1-21, and *Leptolyngbya* sp. NK 1-23 were not identified up to species level, suggesting the possibility of new species/genera. *Leptolyngbya sensu lato* has recently been the focus of several researchers to identify many “*cryptogenus*” as they have rarely distinctive phenotypic and ecological characteristics, yet can be segregated genotypically (Sciuto and Moro, 2016). The unidentified *Leptolyngbya* members from Yunnan hot springs also provided similar scopes for taxonomic novelties. Therefore, it is nevertheless to state that many new potential cyanobacteria can be described from these habitats in the near future. Additionally, these geographically isolated habitats harbor the indigenous thermophilic cyanobacteria (Castenholz, 1969; Miller et al., 2009), which confirmed our findings on the survival of most of the isolates at high temperatures. In the study of phylogeny of cyanobacterial isolates in the present work, the clustering of isolated cyanobacterial species with major OTUs of cyanobacteria under the same subsection reported from 16S rRNA amplicon sequencing analysis of Yunnan hot springs supports the possibility for isolation of major mat-forming strains in culture conditions. However, phylogenetic relationships between all isolated cyanobacteria species and their OTUs showed that many strains could not be isolated in culture conditions. Most of the cyanobacterial strains (especially *Leptolyngbya*) from Yunnan hot springs showed some phylogenetic relationship to thermophilic cyanobacteria in other biogeographically regions, which confirms the presence of cosmopolitan species in the Yunnan hot springs, which were supported by previous findings (Dadheech et al., 2013). It is unlikely that clear patterns of phylogeny and separate clades were observed for *Synechococcus* from hot springs of different countries (Papke et al., 2003). These different patterns of phylogeny and occurrence of endemic cyanobacterial strains in hot springs were supported by the hypothesis given by Papke et al. (2003) that either insufficient time for divergence at the genetic level or the restriction by the presence of dominating cyanobacterial strains in various local niches for evolutionary radiation is responsible. Overall, these findings suggest that geographical isolation of hot springs all over the world with distinct environmental parameters is the main reason for the divergence and endemism of thermophilic cyanobacteria.

In a nutshell, the current work is mainly focused on cyanobacterial census and thermophilic isolates from hot springs. Cyanobacteria are photosynthetic prokaryotes and one of the potential bio-resources for biotechnological applications. They represent a large and diverse group with only a limited history of characterization and exploitation. The application of thermophilic cyanobacteria is still relatively untapped due to the unavailability of novel isolates (unialgal/axenic strains). The isolation, characterization, and bio-prospection of thermophilic cyanobacteria have their own importance because they are the source of many useful temperature-tolerant enzymes and bioactive molecules (Forlani et al., 2008; Nozzi et al., 2013; Ibrahim et al., 2014; Li et al., 2015a). The present study also offers many potential thermophilic cyanobacteria for a detailed study and future biotechnological utilization.

## Conclusion

The study of biodiversity and isolation of potential strains from extreme habitats is a key step for modern research, which not merely describes diversity, but offers the potential taxa for biotechnological exploration. The hot springs located in Tengchong of central-western Yunnan Province is the largest and most intensively studied geothermal field in China. Microbial diversity emphasizing cyanobacterial community structure with isolation of potential taxa is studied using 16S rRNA amplicon sequencing and microscopic approach as well as cultivation-dependent and -independent strategies. The taxonomy and isolation of thermophilic cyanobacteria from hot springs of Yunnan Province open a platform for increasing our understanding of cyanobacterial diversity, their potential, and novelty, which have never been cultivated under laboratory conditions. These strains can be further explored for natural products and temperature tolerance. Overall, the current findings are solid evidence for taxonomy and future exploration of thermotolerant cyanobacteria for biotechnological utilization.

## Data Availability Statement

The data presented in the study are deposited in the NCBI repository, accession numbers SRR11796781–SRR11796799 and MK625304–MK625325.

## Author Contributions

NK, TZ, and XL contributed to the sampling. NK and TZ performed the experiments and drafted the article with contributions from all authors. NK, YZ, SD, and TZ analyzed the data. TZ and XL designed the research and revised the manuscript. All authors contributed to the article and approved the submitted version.

## Conflict of Interest

The authors declare that the research was conducted in the absence of any commercial or financial relationships that could be construed as a potential conflict of interest.

## Publisher’s Note

All claims expressed in this article are solely those of the authors and do not necessarily represent those of their affiliated organizations, or those of the publisher, the editors and the reviewers. Any product that may be evaluated in this article, or claim that may be made by its manufacturer, is not guaranteed or endorsed by the publisher.

## Footnotes

1
https://www.ncbi.nlm.nih.gov/
