# Supplementary material for: Cyanobacterial Community Structure and Isolates From Representative Hot Springs of Yunnan Province, China Using an Integrative Approach
Source: Front Microbiol. 2022 Apr 25;13:872598. doi: 10.3389/fmicb.2022.872598 (PMC9083006; doi:10.3389/fmicb.2022.872598)
Supplement: Supplementary file 1 [file Data_Sheet_1.doc]

Supplementary Tables

***Supplementary Table 1.*** *Distribution pattern of cyanobacteria, green algae and diatoms in different studied hot springs/hot water source of China, based on morphological traits. T. oregonensis is Thermoleptolyngbya oregonensis.*

| Sl. No. | Algal taxa | Hot springs | | | | | | | | | | | | | | | |
| --- | --- | --- | --- | --- | --- | --- | --- | --- | --- | --- | --- | --- | --- | --- | --- | --- | --- |
|  |  | 1 | 2 | 3 | 4 | 5 | 6 | 7 | 8 | 9 | 10 | 11 | 12 | 13 | 14 | 15 | 16 |
| 1 | *Synechococcus* cf. *nidulans* | - | - | + | - | - | - | - | - | - | - | - | - | - | - | - | - |
| 2 | *Synechococcus elongatus* | - | - | - | - | - | - | - | - | - | - | - | - | - | - | - | + |
| 3 | *Gloeocapsa gelatinosa* | + | - | + | - | - | - | - | - | - | - | - | - | - | - | - | - |
| 4 | *Gloeocapsa sanguina* | - | - | + | - | - | - | - | - | - | - | - | - | - | - | - | - |
| 5 | *Cyanothece* sp. | - | - | - | - | - | - | - | - | - | - | - | - | + | - | - | + |
| 6 | *Aphanothece microscopica* | - | - | + | - | - | - | - | - | - | - | - | - | - | - | - | - |
| 7 | *Aphanocapsa thermalis* | + | - | + | - | - | - | - | - | - | - | - | - | - | - | - | - |
| 8 | *Chroococcidiopsis thermalis* | + | - | - | - | - | - | - | - | - | - | - | - | - | - | - | - |
| 9 | *Leptolyngbya copelandii* | - | - | - | + | + | - | - | - | - | - | - | - | - | - | - | - |
| 10 | *Leptolyngbya dangeardii* | - | - | - | - | - | - | - | - | - | - | - | - | + | - | - | - |
| 11 | *Leptolyngbya faveolarum* | + | - | - | - | - |  | - | - | - | - | + | - | - | - | - | - |
| 12 | *Leptolyngbya thermobia* | - | - | - | - | - | + | - | - | - | - | - | - | - | + | - | - |
| 13 | *Leptolyngbya valderiana* | - | - | - | - | - | - | - | - | - | - | + | - | - | - | - | - |
| 14 | *Leptolyngbya* sp. NK 1-10 | - | - | + | - | - | - | - | - | - | - | - | - | - | - | - | - |
| 15 | *Leptolyngbya* sp. NK 1-12 | - | - | + | - | - | - | - | - | - | - | - | - | - | - | - | - |
| 16 | *Leptolyngbya* sp. NK 1-23 | - | - | + | - | - | - | - | - | - | - | - | - | - | - | - | - |
| 17 | *T. oregonensis* | - | - | - | - | - | - | + | + | - | - | + | - | - | - | - | - |
| 18 | *Phormidium ambiguum* | + | - | + | + | - | - | - | - | - | - | - | + | - | - | - | - |
| 19 | *Phormidium terebriforme* | - | - | - | - | - | - | - | - | - | - | - | - | + | + | - | - |
| 20 | *Planktothrix cryptovaginata* | - | - | + | - | - | - | - | - | - | - | - | - | - | - | - | - |
| 21 | *Limnothrix* sp. | - | - | - | - | + | - | - | - | - | - | - | - | - | - | - | - |
| 22 | *Anabaena* sp. NK1-14 | - | - | - | + | - | - | - | - | - | - | - | - | - | - | - | - |
| 23 | *Calothrix* sp. | - | - | + | - | - | - | - | - | - | - | - | - | - | - | - | - |
| 24 | *Chlorogloeopsis fritschii* | - | - | - | - | - | - | - | - | - | - | - | - | - | - | - | + |
| 25 | *Fischerella thermalis* | + | - | - | - | - | - | - | - | - | - | - | - | - | - | - | - |
| 26 | *Fischerella* sp. NK 1-16 | - | - | - | - | - | - | - | - | - | + | - | - | - | - | - | - |
| 27 | *Fischerella* sp. NK 1-20/21 | - | - | - | - | - | - | - | - | - | - | - | + | - | - | - | + |
| 28 | *Stigonema* sp. | - | + | - | - | - | - | - | - | - | - | - | - | - | - | - | - |
| 29 | *Chlorella* sp. | + | + | - | - | - | - | - | - | + | - | - | - | - | - | - | - |
| 30 | *Pinnularia* sp. | - | - | - | - | - | - | - | - | - | - | - | - | - | - | + | - |
| 31 | *Gomphonema* sp. | - | - | - | - | - | - | - | - | - | - | - | - | - | - | + | - |
| 32 | *Cyclotella* sp. | - | - | - | - | - | - | - | - | - | - | - | - | - | - | + | - |
| 33 | *Navicula* sp. | - | - | - | - | - | - | - | - | - | - | - | - | - | - | + | - |
| 34 | *Achnanthes* sp. | - | - | + | - | - | - | - | - | + | - | - | - | - | + | + | - |
| 35 | *Luticola* sp. | - | - | - | + | - | - | - | - | - | - | - | - | - | - | - | - |
| 36 | *Nitzschia* sp. | + | - | - | - | - | - | - | - | - | - | - | - | - | - | - | - |
| 37 | *Melosira* sp. | - | - | - | - | - | - | - | - | - | - | - | - | - | + | - | - |
| 38 | *Rhopalodia* sp. | - | - | - | + | - | - | - | - | - | - | - | - | + | + | - | - |
| 39 | *Sellaphora* sp. | - | - | - | + | - | - | - | - | - | - | - | - | - | - | - | - |
| Total Genera | | 8 | 2 | 10 | 6 | 2 | 1 | 1 | 1 | 2 | 1 | 2 | 2 | 4 | 5 | 5 | 4 |
| Total species | | 8 | 2 | 12 | 6 | 2 | 1 | 1 | 1 | 2 | 1 | 3 | 2 | 4 | 5 | 5 | 4 |

**Supplementary Table 2.** Cyanobacterial OTUs from the hot spring mat of sampling areas in this study and their percentage similarity with closest relative in NCBI database with morphological subsection information.

| Sl. No. | OTU_ID | Subsection | Genera | BLAST % |
| --- | --- | --- | --- | --- |
|  |  |  |  |  |
| 1 | OTU2 | Subsection III | Uncultured Oscillatoriales | 98% |
| 2 | OTU5 | Subsection III | *Phormidium autumnale* | 99% |
| 3 | OTU18 | Subsection III | Pseudanabaenaceae cyanobacterium | 91% |
| 4 | OTU53 | Subsection III | *Leptolyngbya* sp. | 99% |
| 5 | OTU33 | Subsection III | Uncultured Oscillatoriales | 98% |
| 6 | OTU79 | Subsection IV | *Tolypothrix* sp. | 100% |
| 7 | OTU24 | Subsection III | Uncultured *Oscillatoriales* | 98% |
| 8 | OTU34 | Subsection III | *Leptolyngbya* sp. | 100% |
| 9 | OTU54 | Subsection III | *Leptolyngbya* sp. | 100% |
| 10 | OTU43 | Subsection II | Uncultured Chroococcales | 96% |
| 11 | OTU77 | Subsection II | *Chroococcidiopsis cubana* | 99% |
| 12 | OTU203 | Subsection III | *Leptolyngbya* sp. | 97% |
| 13 | OTU45 | Subsection II | *Pleurocapsa*sp.*/Chroococcopsis*sp. | 99% |
| 14 | OTU61 | Subsection III | *Planktothricoides raciborskii* | 98% |
| 15 | OTU131 | Subsection III | *Arthronema africanum* | 97% |
| 16 | OTU68 | Subsection III | *Microcoleus cf. vaginatus* | 99% |
| 17 | OTU111 | Subsection I | *Synechococcus* sp. | 98% |
| 18 | OTU62 | Subsection I | *Thermosynechococcus elongatus* | 100% |
| 19 | OTU98 | Subsection III | Oscillatoriales cyanobacterium | 98% |
| 20 | OTU103 | Subsection I | *Chroococcidiopsis* sp. | 100% |
| 21 | OTU76 | Subsection III | *Leptolyngbya* sp. | 100% |
| 22 | OTU120 | Subsection III | *Leptolyngbya* sp. | 98% |
| 23 | OTU147 | Subsection III | *Leptolyngbya ramosa* | 99% |
| 24 | OTU162 | Subsection IV | *Scytonema hofmanni* | 99% |
| 25 | OTU1481 | Subsection III | *Leptolyngbya* sp. | 98% |
| 26 | OTU133 | Subsection I | *Gloeocapsa* sp. | 99% |
| 27 | OTU213 | Subsection III | *Leptolyngbya copelandii* | 100% |
| 28 | OTU1082 | Subsection II | Uncultured *Chroococcidiopsis* sp. | 99% |
| 29 | OTU1491 | Subsection IV | *Calothrix* sp. | 99% |
| 30 | OTU1242 | Subsection III | *Phormidium murrayii* | 98% |
| 31 | OTU159 | Subsection III | *Cf. Leptolyngbya* sp. | 99% |
| 32 | OTU1503 | Subsection II | *Xenococcus* sp. | 99% |
| 33 | OTU609 | Subsection III | *Nodosilinea* sp.*/Leptolyngbya* sp. | 99% |
| 34 | OTU625 | Subsection III | *Leptolyngbya boryana* | 99% |
| 35 | OTU851 | Subsection III | *Leptolyngbya* sp. | 99% |
| 36 | OTU602 | Subsection II | *Chondrocystis* sp. | 99% |
| 37 | OTU164 | Subsection V | *Iphinoe* sp. | 99% |
| 38 | OTU663 | Subsection II | *Pleurocapsa minor* | 99% |
| 39 | OTU628 | Subsection III | *Arthronema africanum* | 99% |
| 40 | OTU888 | Subsection III | Uncultured *Leptolyngbya* sp. | 98% |
| 41 | OTU1276 | Subsection I | *Aphanothece hegewaldii* | 97% |
| 42 | OTU722 | Subsection III | *Leptolyngbya* sp. | 98% |
| 43 | OTU911 | Subsection III | Leptolyngbyaceae cyanobacterium | 98% |
| 44 | OTU558 | Subsection III | Leptolyngbyaceae cyanobacterium | 95% |
| 45 | OTU718 | Subsection V | *Stigonema elegans* | 99% |
| 46 | OTU328 | Subsection I | Uncultured *Synechococcus* | 99% |
| 47 | OTU1042 | Subsection I | *Aphanothece hegewaldii* | 98% |
| 48 | OTU708 | Subsection III | *Leptolyngbya* sp. | 99% |
| 49 | OTU577 | Subsection III | Leptolyngbyaceae cyanobacterium | 97% |
| 50 | OTU952 | Subsection III | Oscillatoriales cyanobacterium | 99% |
| 51 | OTU1261 | Subsection III | *Wilmottia murrayi/Phormidium* sp. | 97% |
| 52 | OTU1223 | Subsection III | *Ancylothrix terrestris* | 100% |
| 53 | OTU1257 | Subsection III | *Wilmottiam urrayi/Phormidium* sp. | 99% |
| 54 | OTU1522 | Subsection V | *Stigonema* sp. | 96% |
| 55 | OTU919 | Subsection III | *Leptolyngbya subtilis* | 99% |
| 56 | OTU1254 | Subsection III | *Wilmottia* sp.*/Phormidium* sp. | 99% |
| 57 | OTU298 | Subsection V | Thermophilic cyanobacterium | 99% |
| 58 | OTU1266 | Subsection I | *Chroococcus* sp. | 96% |
| 59 | OTU931 | Subsection II | *Chroococcidiopsis* sp. | 98% |
| 60 | OTU1250 | Subsection III | *Leptolyngbya* sp. | 97% |
| 61 | OTU307 | Subsection I | *Synechococcus* sp. | 95% |
| 62 | OTU1273 | Subsection III | *Jaaginema geminatum* | 99% |
| 63 | OTU982 | Subsection III | *Leptolyngbya* sp. | 96% |
| 64 | OTU848 | Subsection III | *Leptolyngbya* sp. | 96% |
| 65 | OTU287 | Subsection III | *Phormidium* sp. | 94% |
| 66 | OTU1319 | Subsection III | *Microcoleus* sp. | 99% |
| 67 | OTU689 | Subsection III | *Leptolyngbya* sp. | 99% |
| 68 | OTU1619 | Subsection V | *Chlorogloeopsis fritschii* | 96% |
| 69 | OTU617 | Subsection III | *Leptolyngbya* sp. | 95% |
| 70 | OTU904 | Subsection III | *Leptolyngbya foveolarum* | 96% |
| 71 | OTU721 | Subsection IV | *Nostoc* sp. | 99% |
| 72 | OTU1483 | Subsection III | *Leptolyngbya* sp. | 97% |
| 73 | OTU709 | Subsection III | *Leptolyngbya* sp. | 99% |
| 74 | OTU1520 | Subsection IV | *Calothrix fusca* | 95% |
| 75 | OTU691 | Subsection II | *Chroococcidiopsis* sp. | 100% |
| 76 | OTU1343 | Subsection II | *Pseudocapsa* sp. | 95% |
| 77 | OTU1781 | Subsection III | *Pseudanabaena* sp. | 95% |
| 78 | OTU1510 | Subsection II | *Pleurocapsa* sp. | 98% |
| 79 | OTU626 | Subsection III | *Leptolyngbya* sp. | 98% |
| 80 | OTU1542 | Subsection I | *Cyanobium* sp. | 99% |
| 81 | OTU667 | Subsection III | *Microseira wollei/Phormidium* sp. | 95% |
| 82 | OTU883 | Subsection II | *Chroococcidiopsis bourrellyana* | 97% |
| 83 | OTU961 | Subsection III | Uncultured *Leptolyngbya* sp. | 90% |
| 84 | OTU925 | Subsection III | *Leptolyngbya* sp. | 99% |
| 85 | OTU1502 | Subsection I | *Oscillatoria* sp. | 91% |
| 86 | OTU1455 | Subsection II | *Chroococcidiopsis* sp. | 95% |
| 87 | OTU1269 | Subsection III | *Phormidium autumnale* | 99% |
| 88 | OTU521 | Subsection V | *Mastigocladus laminosus* | 99% |
| 89 | OTU1579 | Subsection I | *Aphanothece sacrum* | 95% |
| 90 | OTU1019 | Subsection II | *Gloeocapsopsis* sp. | 99% |
| 91 | OTU1240 | Subsection III | *Phormidium autumnale* | 99% |
| 92 | OTU1183 | Subsection II | Uncultured *Chroococcidiopsis* sp. | 99% |
| 93 | OTU1328 | Subsection III | Oscillatoriaceae cyanobacterium | 98% |
| 94 | OTU1275 | Subsection II | *Chroococcidiopsis* sp. | 97% |
| 95 | OTU1234 | Subsection III | *Microcoleus* sp. | 97% |
| 96 | OTU565 | Subsection III | Oscillatoriales cyanobacterium | 99% |
| 97 | OTU1339 | Subsection II | Uncultured *Chroococcidiopsis* sp. | 95% |
| 98 | OTU972 | Subsection I | *Chroococcus* sp. | 96% |
| 99 | OTU1284 | Subsection III | *Leptolyngbya* sp. | 96% |
| 100 | OTU639 | Subsection I | *Gloeocapsa* sp. | 97% |
|  |  |  |  |  |

**Supplementary Table 3.** Comparison of isolated cyanobacteria and 16S rRNA sequencing study.

| Codes | Isolated cyanobacteria | | 16S rRNA analysis |
| --- | --- | --- | --- |
|  | Species no. | % Similarity (NCBI blast) & Subsection | Major OUT & Subsection |
|  |  |  |  |
| Ds001 | NK1-7 | *Lyngbya* sp. (KX263924) 100%, Subsection III | OTU5:Subsection III |
| Hp002 | - | - | OTU7:(bacteria)  OTU722:Subsection III |
| Hp003 | NK1-12 | Oscillatoriales cyanobacterium (KU557673) 99%, Subsection III | OTU5:Subsection III |
|  | NK1-10 | *Leptolyngbya* sp. (LC319755) 100%, Subsection III |  |
|  | NK1-11 | *Planktothrixpseudagardhii* (JQ894510) 95%, Subsection III |  |
|  | NK1-23 | *Leptolyngbya* sp. (KM384748) 96%, Subsection III |  |
| Rh004 | NK1-13 | *Leptolyngbya* sp. (KM438181) 97%, Subsection III | OTU8:(bacteria)  OTU2:Uncultured cyanobacterium |
|  | NK1-14 | *Anabaena* sp. (HM235817) 98%, Subsection IV | OTU1491:Subsection IV |
| Rh005 | - | - | OTU2:Subsection III |
| Rh006 | NK1-25 | *Leptolyngbya*sp. (LC319755) 100%, Subsection III | OTU3:bacteria  OTU24:Subsection III |
| Rh007 | NK1-24 | *Chlorogloeopsisfritschii* (KT807481) 100%, Subsection V | OTU4:bacteria  OTU1619:Subsection V |
| Rh008 | NK1-15 | *Leptolyngbya*sp. (AP017367) 99%, Subsection III | OTU18:Subsection III |
| Rh009 | - | - | No cyanobacteria |
| Qs010 | NK1-16 | *Fischerella*sp. (AB093491) 99%, Subsection V | OTU27 (bacteria)  No cyanobacteria |
| Blz011 | NK1-22 | *Leptolyngbya*sp. (AP017367) 99%, Subsection III | OTU20:bacteria  OTU54:Subsection III |
| Blz012 | NK1-20 | *Fischerella* sp. (DQ786171) 100%, Subsection V | OTU33:Subsection III  OTU298, Subsection V |
| Blz013 | NK1-27 | *Leptolyngbya* sp. (MF405380) 100%, Subsection III | OTU2:Subsection III |
| Blz014 | NK1-28 | *Leptolyngbyathermobia* (KM376990) 100%, Subsection III | OTU2, Subsection III |
| Blz015 | - | - | No cyanobacteria |
| Blz016 | NK1-26 | *Leptolyngbya* sp. (AP017367) 100%, Subsection III | OTU8:bacteria  OTU24:Uncultured cyanobacterium |
