# Supplementary material for: Cyanobacterial Community Structure and Isolates From Representative Hot Springs of Yunnan Province, China Using an Integrative Approach
Source: Front Microbiol. 2022 Apr 25;13:872598. doi: 10.3389/fmicb.2022.872598 (PMC9083006; doi:10.3389/fmicb.2022.872598)

Supplementary Figures

Supplementary Figure 1 Anions, cations and conductivity analysis of the water samples from hot spring in this study.

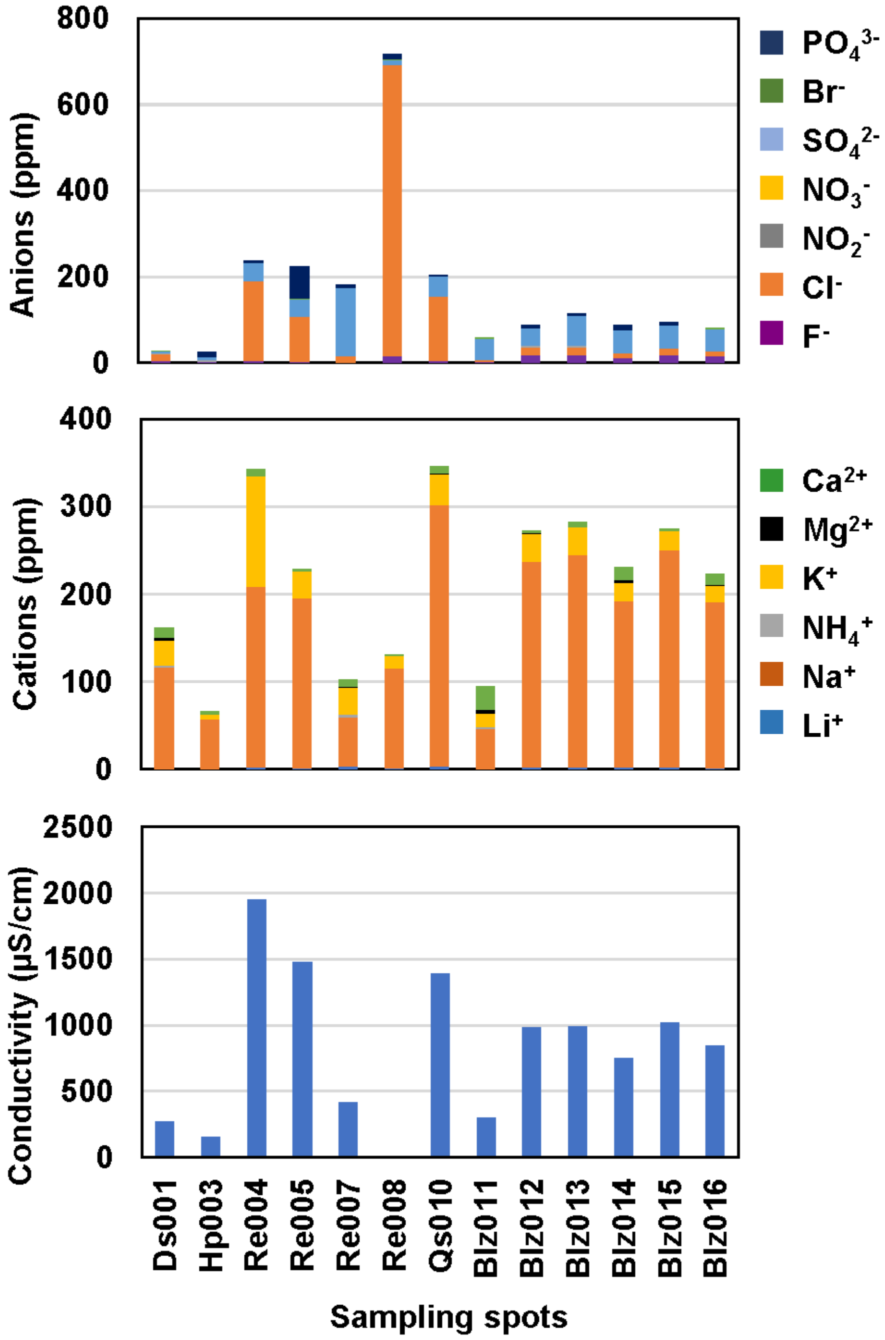

Supplementary figure 2 OTU Venn diagram illustrating distribution of microbial OTUs among the four groups of temperature range. T indicates the temperatures of the water samples or the hot spring water temperatures of other sample types. T30-50 (Group 1: 30–50°C), T50-70 (Group 2: 50–70°C), T70-80 (Group 3: 70–80°C), T80-90 (Group 4: 80–90°C). The Venn diagram was generated using the OmicStudio tools (https://www.omicstudio.cn/tool).

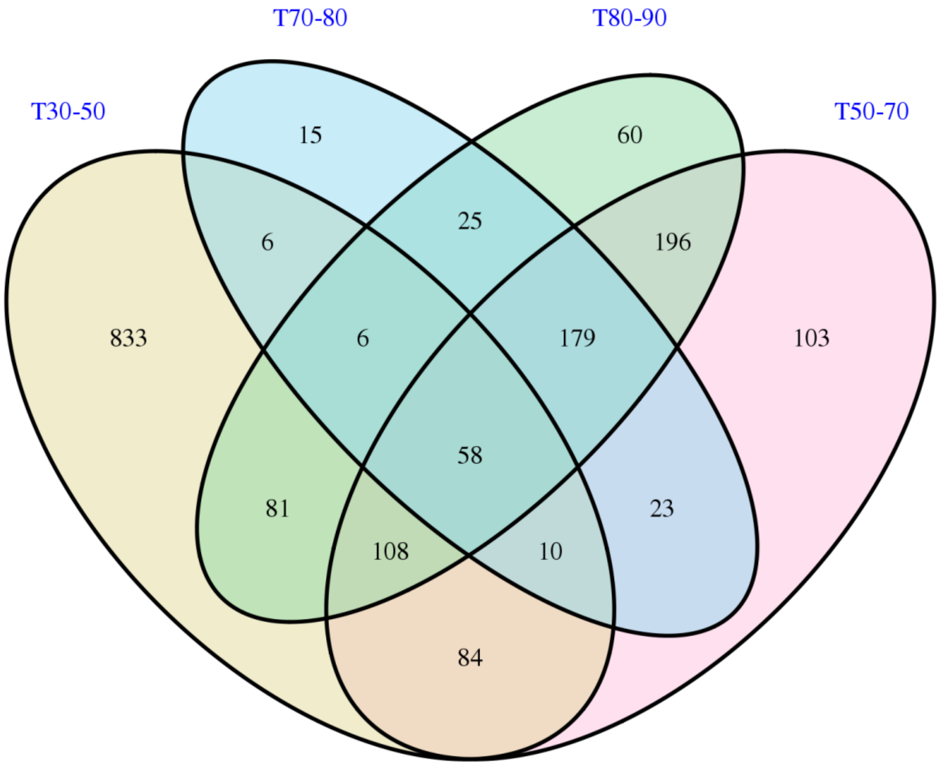

Supplementary figure 3 Chao1 estimates of OTU richness in the different groups of thermal springs. T indicates the temperatures of the water samples or the hot spring water temperatures of other sample types. T30-50 (Group 1: 30–50°C), T50-70 (Group 2: 50–70°C), T70-80 (Group 3: 70–80°C), T80-90 (Group 4: 80–90°C).

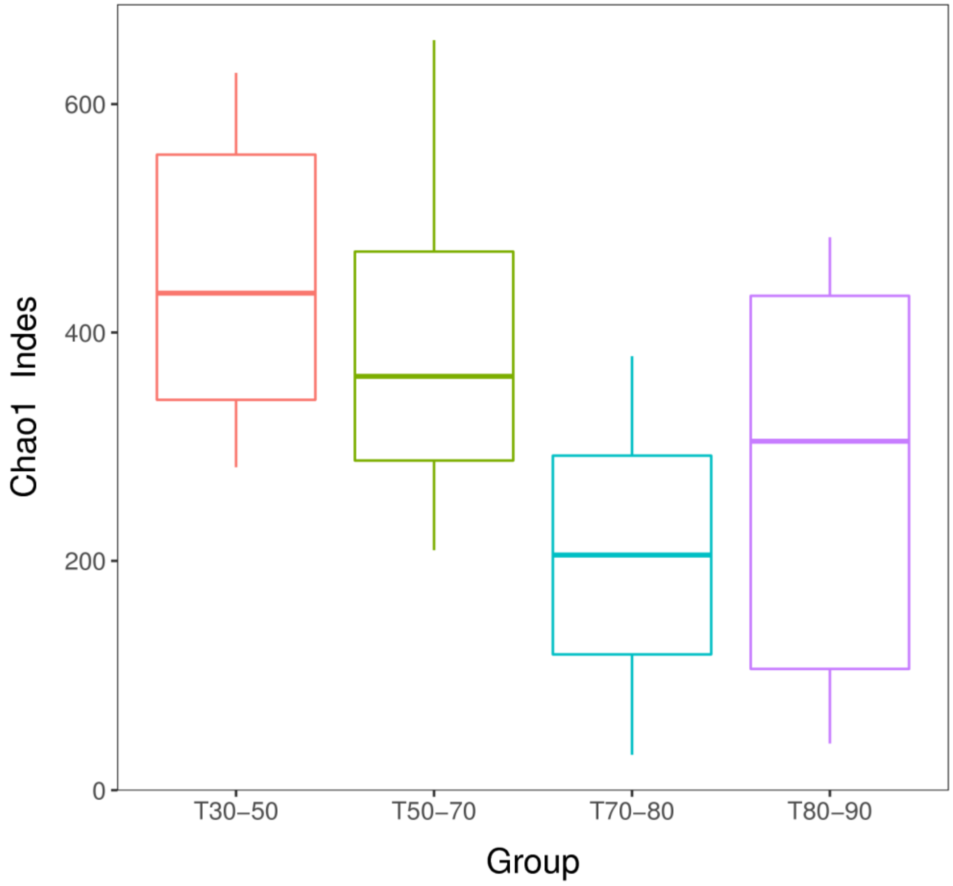

Supplementary figure 4 The microbial OTU abundance clustering heat map. The rows and columns represent the OTU IDs and the sampling sites, respectively. The OTU clustering tree is in the left. The value of each square color of the middle heat map corresponds to the relative abundance of each row of OTU.

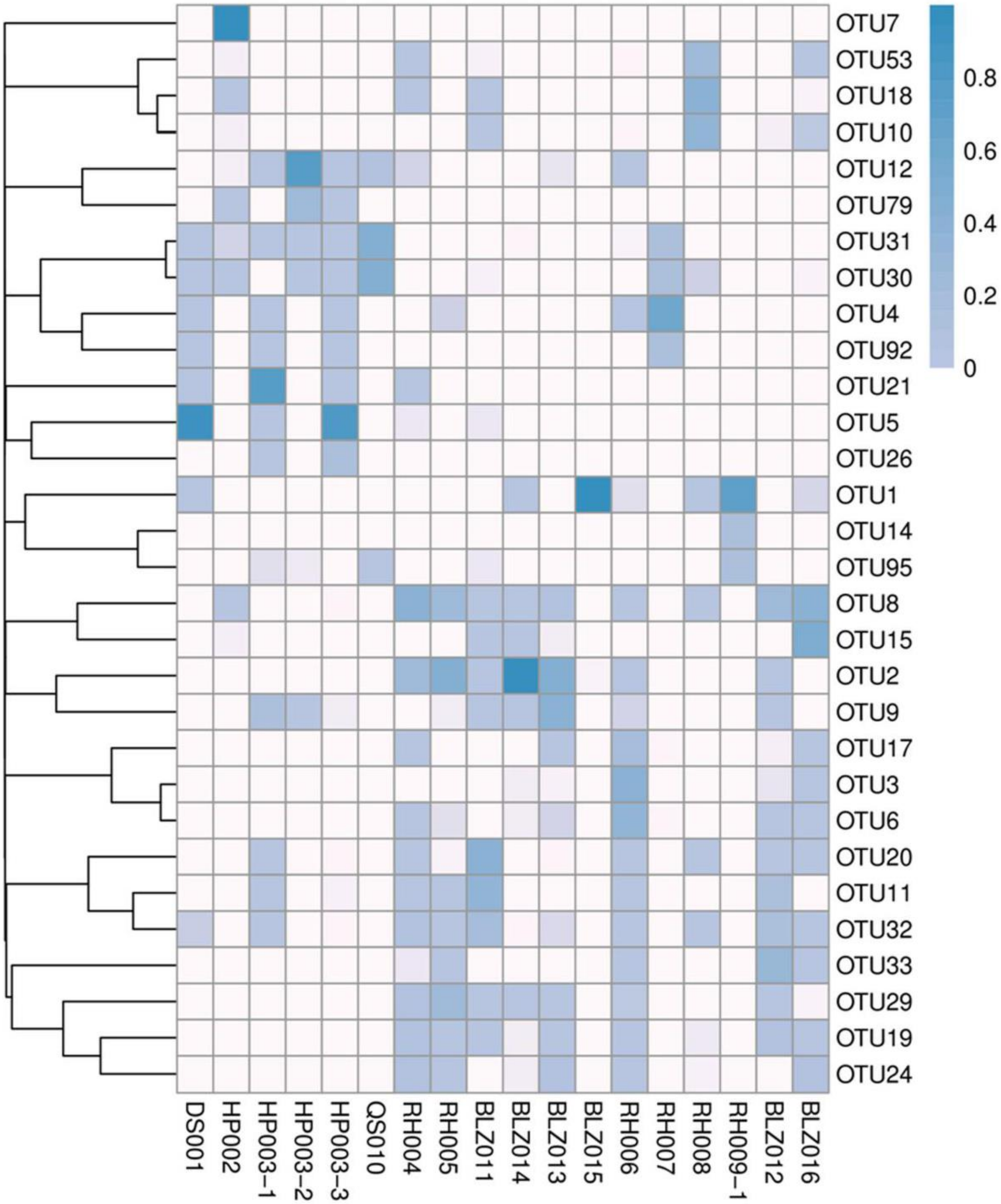

Supplementary figure 5 Microbial community composition basing on the sampling sites (A) and temperature groups (B). Stacked column graph representing the relative distribution of the dominant phyla in the different mats. Top30 is plotted as a histogram.

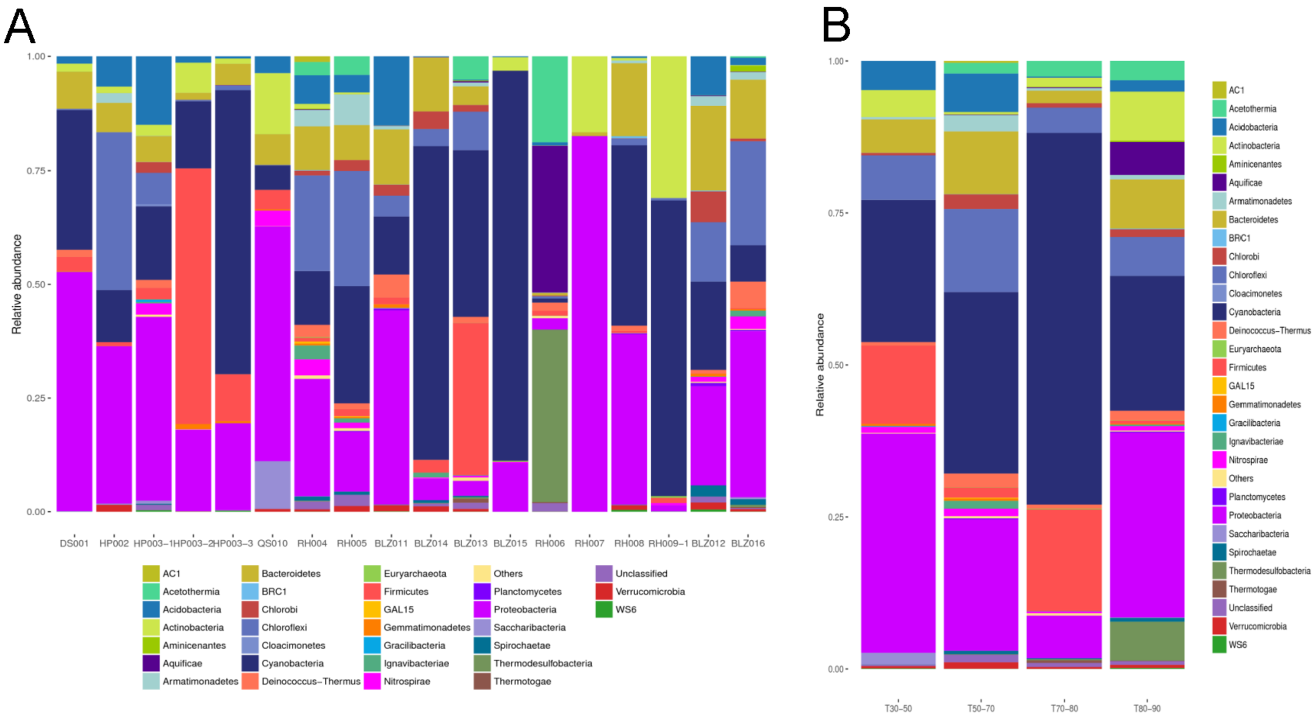

Supplementary figure 6 Other associated algal members. A. *Aphanocapsa thermalis*, B. *Cyanothece sp.*, C. *Aphanothece microscopica*, D. *Asterocapsa divina*, E. *Gloeocapsa gelatinosa*, F. *Gloeocapsa sanguinea*, G. *Chroococcidiopsis thermalis*, H. *Synechococcus elongatus*, I. *Limnothrix* sp., J. *Phormidium ambiguum*, K. *Phormidium terebriforme*, L. *Calothrix* sp., M. *Stigonema* sp.

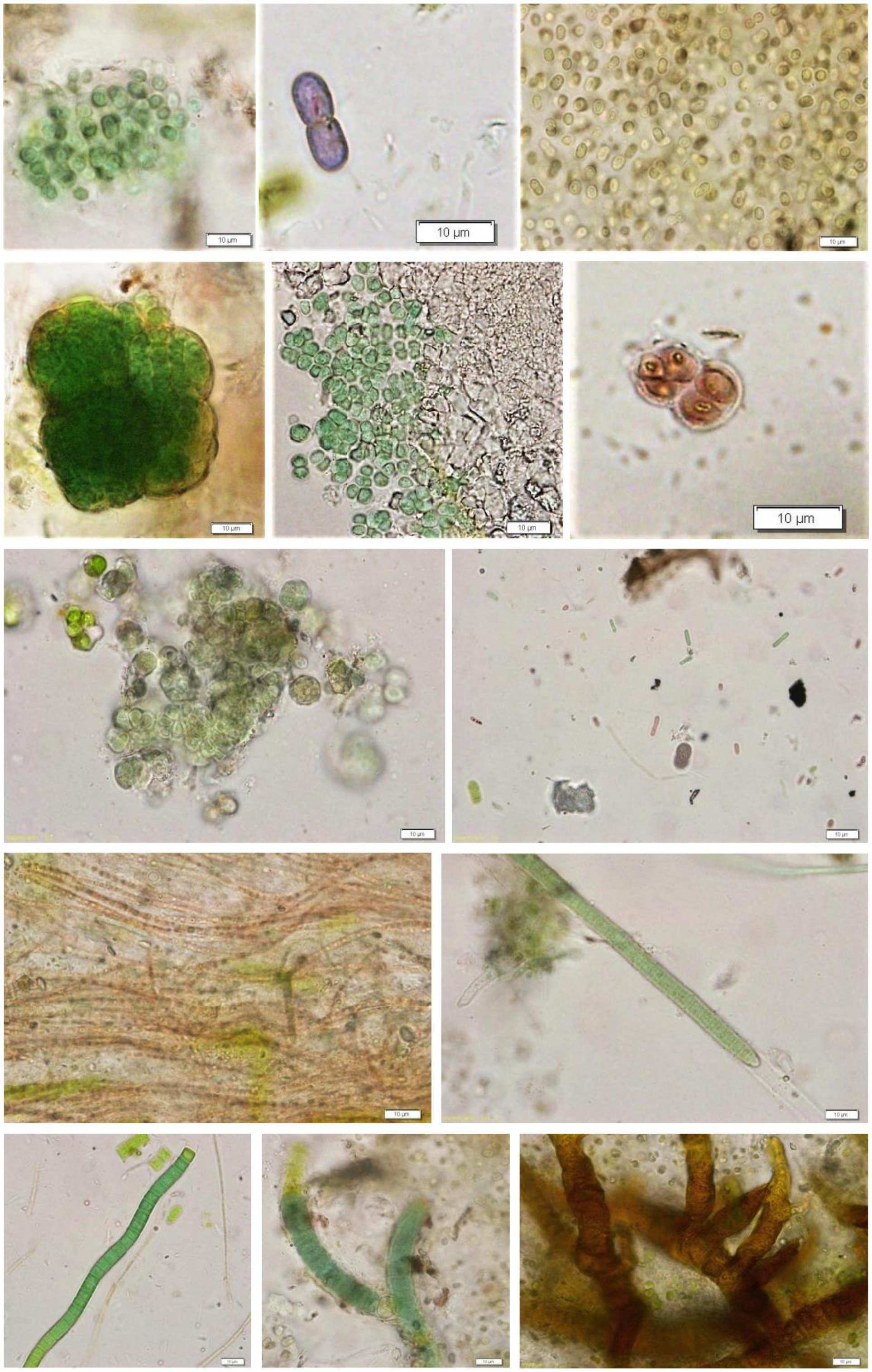

Supplementary figure 7 Phylogenetic relationships (Neighbor-Joining) between the 16S rRNA gene sequences of isolated cyanobacteria and major OTUs obtained from high-throughput sequencing (including bacteria and cyanobacteria) of hot springs in this study. The number near the node represents the bootstrap value. *Gloeobacter violaceus* PCC 7421 (NR 074282) and *E.coli* (J01695) were taken as the references for cyanobacteria and bacteria respectively.

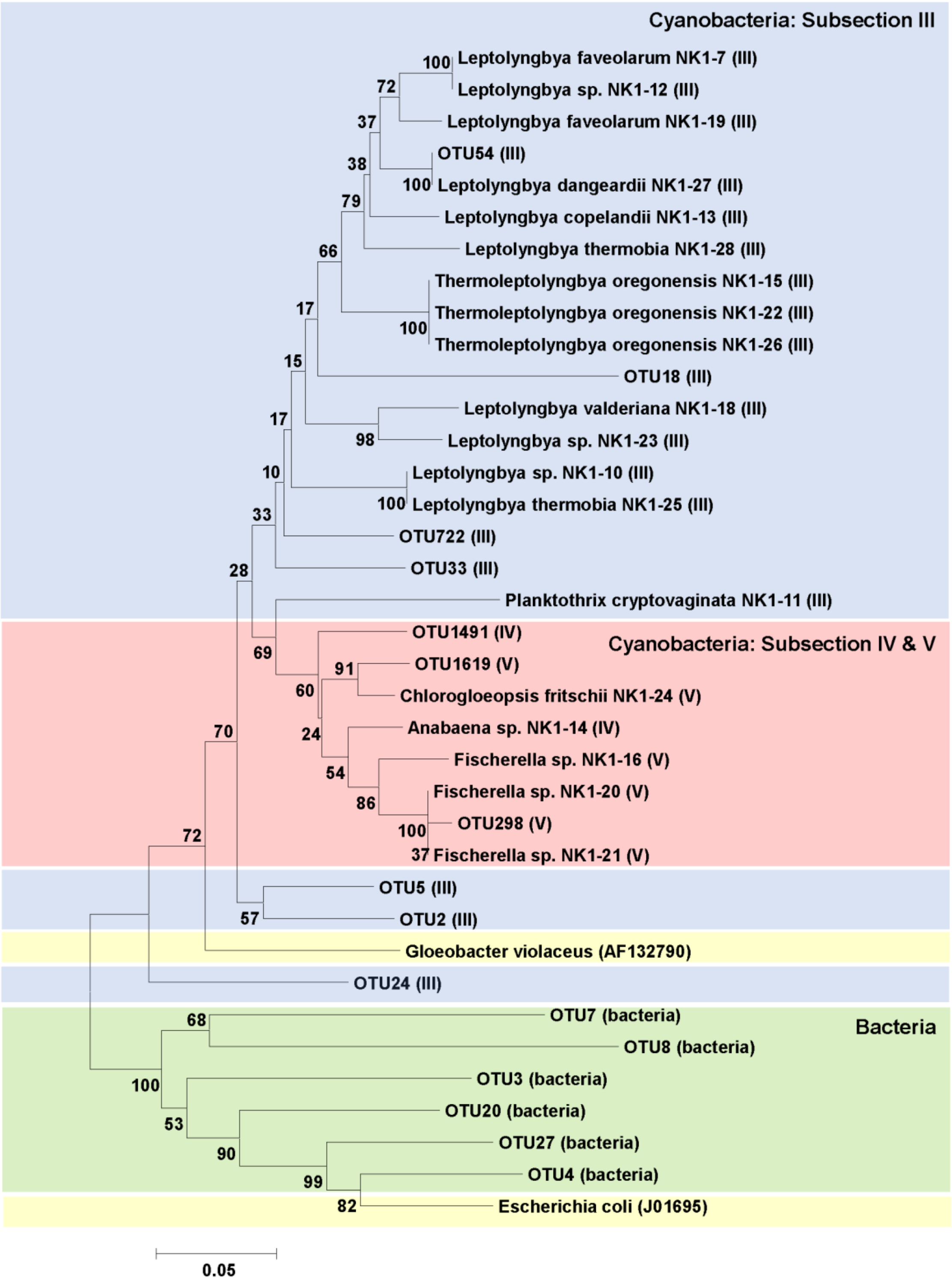

Supplementary figure 8 Phylogenetic relationships (Neighbor-Joining) between the 16S rRNA gene sequences of isolated cyanobacteria species and all cyanobacterial OTUs of 16S rRNA gene amplicons analysis of hot springs in this study. Isolated cyanobacteria were indicated in red color. The number near the node represents the bootstrap value. Subsection was mentioned in brackets. *Gloeobacter violaceus* PCC 7421 (AF132790) was used as an out-group.

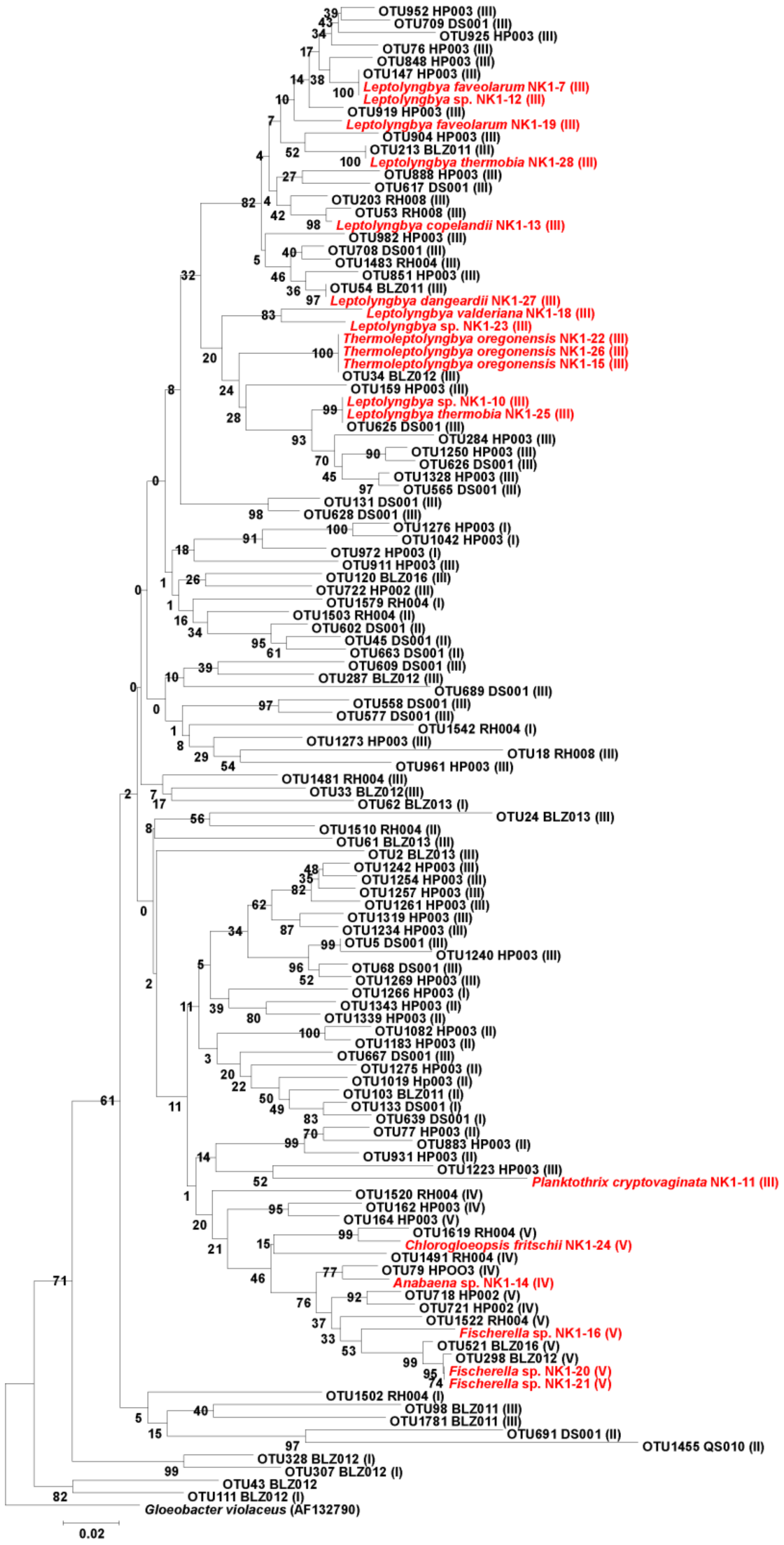

Supplement: Supplementary file 2 [file Data_Sheet_2.pdf]
